# Development of an Olfactory Receptor (OR5K1)-Based Biosensor for Pyrazine Detection in Foods

**DOI:** 10.3390/foods15183355

**Published:** 2026-09-21

**Authors:** Lele Zhang, Yan Ping Chen, Suet Yee Tan, Zhiping Xie, Xichang Wang, Yuan Liu

**Affiliations:** 1 College of Food Science and Technology, Shanghai Ocean University, Shanghai 200240, China; 15553302832@163.com; 2 School of Agriculture and Biology, Shanghai Jiao Tong University, Shanghai 200240, China; 3 School of Health Science and Engineering, University of Shanghai for Science and Technology, Shanghai 200093, China; 4 State Key Laboratory of Microbial Metabolism and Joint International Research Laboratory of Metabolic & Developmental Sciences, School of Life Sciences and Biotechnology, Shanghai Jiao Tong University, Shanghai 200240, China; 5 School of Food Science and Engineering, Ningxia University, Yinchuan 750021, China

**Keywords:** olfactory biosensor, OR5K1, pyrazine, ZIF-8@SWCNT/Ti_3_C_2_ MXene, molecular docking

## Abstract

Pyrazines are the key odorants in food contributing to baked and nutty aromas. In order to improve practicality, a novel electrochemical olfactory biosensor for pyrazine analysis was developed using an olfactory receptor (OR5K1) as the recognition element and AuNPs-PB/ZIF-8@SWCNT/Ti_3_C_2_ MXene as the sensing matrix. The sensing application and molecular recognition mechanism of OR5K1 toward pyrazines were explored using molecular docking and in silico site-directed mutagenesis. We explored the sensing application and molecular recognition mechanism of OR5K1 toward pyrazines using molecular docking and in silico site-directed mutagenesis. The sensor achieved a linear detection range of 10^−14^ to 10^−9^ M with a low detection limit of 10^−14^ M. The biosensor exhibited significantly higher current responses toward pyrazine compounds compared to interfering substances, including ethanol, hexanal, acetone, and phenol, demonstrating excellent selectivity, and retained 79% of its initial signal after 12 days of storage, indicating good stability. The change in the reduction peak current (ΔI) in the presence of pyrazine was used as an analytical signal. When applied to four malt samples (pilsner, munich, crystal, and caramel malts), the biosensor showed ΔI responses ranging from 4.6 ± 0.2 µA to 108 ± 6 µA, which corresponded well with the total pyrazine contents determined by GC-TOF/MS (0.092–0.391 mg/kg), with a correlation coefficient of 0.96. Molecular docking revealed binding energies ranging from −3.9 to −6.0 kcal/mol, suggesting spontaneous interactions between OR5K1 and pyrazines, with Leu14, Met81, Asn84, Phe17, Phe85, and Lys90 identified as potential key residues and hydrogen bonds, hydrophobic interactions, and π−π stacking as primary driving forces. This work provides a sensitive and selective biosensor for pyrazine detection, and the elucidated recognition mechanism offers a molecular basis for understanding roasted aroma perception, supporting applications in food quality control and flavor analysis. Collectively, this study offers new insights for designing olfactory receptor-based electrochemical biosensors and facilitates future exploration of food aroma–receptor interaction mechanisms.

## 1. Introduction

Pyrazines (1,4-diazabenzene) are a class of nitrogen-containing heterocyclic compounds with a pyrazine core structure [1]. They are widely distributed in various natural foods, including coffee, beer malt, meat, and Chinese baijiu. Owing to extremely low odor thresholds, pyrazines serve as key aroma-active compounds, and their formation levels are commonly employed as an indicator for assessing the sensory quality of foods [2]. They are predominantly generated by the Maillard reaction and exhibit pleasant baked, roasted, and nutty flavor notes [3]. Beyond their roles in food flavor, pyrazines are also well-recognized semiochemicals [4], synthesized in living organisms and exhibiting a certain degree of persistence in the environment [5]. Thus, many pyrazines have been reported to mediate chemical signal transmission between individuals of different species, such as inducing aversive and fear-associated responses [6,7].

Olfaction begins with the specific binding of odorant molecules to olfactory receptors (ORs), which initiates a signaling cascade that the brain interprets as smell [8]. The olfactory receptor OR5K1 has recently been characterized as an OR specialized for detecting pyrazines. After identifying 1100 compound odor activators for 273 potentially functional human ORs, Wilkin et al. revealed 2,5-dimethylpyrazine as a specific agonist to OR5K1 [9]. Among 616 human OR alleles, only OR5K1 and its close homologs responded strongly to 2,3,5-trimethylpyrazine, demonstrating high selectivity toward pyrazines [10]. OR5K1, located on chromosome 3 (3q11.2), ranks among the most abundant olfactory receptors in humans (approximately 6%) [11]. It belongs to the class A rhodopsin-like family of the G protein-coupled receptor superfamily and possesses the characteristic seven-transmembrane architecture. Unlike non-sensory class A GPCRs, OR5K1 exhibits low sequence homology and remains largely an orphan receptor, with only a limited number of recognized targets identified [12]. This protein is predominantly expressed in olfactory sensory neurons, where ligand binding triggers conformational changes that activate G proteins, leading to cAMP elevation, channel opening, and, ultimately, action potentials being transmitted to the olfactory bulb, enabling perception of the corresponding odor [13].

High-sensitivity and selective detection of pyrazine compounds have significant practical application value. Conventional detection methods mainly employ gas chromatography systems, such as GC-MS and GC-TOF/MS, which enable accurate qualitative and quantitative detection of pyrazines. But, this equipment is costly and lacks portability, and the operation is complex and requires specialized personnel [14]. Commercially available electronic noses are portable. Gas sensors based on conductive nanomaterials have achieved rapid detection of odor compounds [15]. Since their detection mechanisms rely on non-specific interactions between materials and odor molecules, the selectivity of these sensors lacks specificity [16]. In recent years, various nanomaterials have been extensively explored to enhance the performance of electrochemical biosensors. Metal–organic frameworks (MOFs), such as zeolitic imidazolate framework−8 (ZIF−8), are crystalline porous materials composed of metal ions/clusters coordinated to organic ligands, offering exceptional thermal stability, ultrahigh porosity, and large specific surface areas, making them ideal carriers for enzyme immobilization and signal amplification [17,18]. Wang et al. synthesized in situ silver nanoparticles (AgNPs) on a cobalt-based 2-methylimidazole zeolitic imidazolate framework (ZIF-67). They combined the large specific surface area, resistance to particle aggregation, and excellent biocompatibility of ZIF-67 with the catalytic properties of silver nanoparticles [19]. Gold nanoparticles (AuNPs) facilitate efficient electron transfer and provide biocompatible interfaces for receptor immobilization via Au–S covalent bonds. AuNPs, owing to their unique electrochemical properties and favorable biocompatibility, have been widely applied in biosensor fabrication, point-of-care diagnostics, food safety monitoring, and healthcare-related fields [20,21,22]. Single-walled carbon nanotubes (SWCNTs) possess a large specific surface area and excellent electrical conductivity, which significantly improve electron transport efficiency when incorporated into composite materials [23]. Ti_3_C_2_ MXene, a two-dimensional transition metal carbide, was selected due to its metallic conductivity, hydrophilicity, large interlayer spacing, and abundant surface functional groups (–O, –OH, –F), which not only amplify electrochemical sensing signals but also provide a favorable microenvironment for biomolecule immobilization and facilitate rapid electron transfer [24,25]. In this composite, ZIF-8 provides a high surface area for dense receptor loading, SWCNTs serve as conductive pathways to overcome the poor conductivity of ZIF-8, MXene enhances the electrode conductivity and promotes uniform film formation, and AuNPs improve electron transfer while ensuring stable receptor immobilization. The synergistic combination of these nanomaterials offers a promising strategy for constructing high-performance olfactory biosensors with improved sensitivity, selectivity, and stability. Biosensors based on olfactory receptors have emerged as promising candidates for odor analysis due to their high sensitivity and specificity [15,26]. The synergistic effects of composite nanomaterials combined with olfactory receptors provide an innovative pathway for high-performance electrochemical biosensors.

To study the structure–activity relationships of odorants, computer modeling and chemoinformatics have been increasingly applied based on OR activity [12,27,28]. By analyzing OR sequences and constructing computational models, several key amino acid residues potentially involved in odorant recognition were identified. Upon odorant binding, ORs may undergo conformational changes analogous to those observed in other GPCRs, such as the shift of the third transmembrane helix, which exposes the DRY motif to facilitate G protein activation. This approach enables the identification of putative binding pockets and highlights critical residues that may govern receptor specificity. Collectively, these methodologies yield insights into receptor–ligand interactions, including binding modes, binding sites, binding affinity, thermodynamics, and kinetics, as well as the stability and conformational dynamics of receptor–ligand complexes [29]. Molecular docking is a powerful tool to analyze the atomic-level interactions between molecules and olfactory receptors, which can be applied to investigate the formation mechanisms of aroma compounds [30,31,32]. Current research on constructed ligand-binding site models of OR5K1 primarily relies on artificial intelligence modeling and homology modeling approaches, thus inferring its binding patterns with odor molecules [12]. These studies on binding mechanisms facilitate the screening of aroma molecules with specific roasted aroma characteristics and guide subsequent roasted aroma regulation [33]. However, to our knowledge, the binding mechanism between pyrazine molecules and olfactory receptor OR5K1 has not yet been reported.

The objectives of this study are to (i) propose a novel olfactory biosensor based on OR5K1, (ii) apply the constructed biosensor for detecting pyrazine compounds in real samples, and (iii) investigate the interaction mechanism between OR5K1 and pyrazine through molecular simulations and in silico site-directed mutagenesis. This work fills the gap in the “targeted identification” of pyrazine compounds, elucidates the molecular recognition mechanisms of pyrazine-related roasted aromas, and guides subsequent in vitro functional experiments.

## 2. Materials and Methods

### 2.1. Chemicals and Analytical Standards

Pyrazine, 2,3,5-trimethylpyrazine (TMP), 2,3,5,6-tetramethylpyrazine (TTMP), 2,6-dimethylpyrazine (2,6-DMeP), 2,3-dimethylpyrazine (2,3-DMeP), and 2,5-dimethylpyrazine (2,5-DMeP) were purchased from Adamas Reagent Co., Ltd. (Shanghai, China). Methanol, polyvinylpyrrolidone (PVPP), citric acid, anhydrous sodium sulfate, sodium chloride, disodium hydrogen phosphate, zinc nitrate, 2-methylimidazole, and DL-dithiothreitol (DTT) were purchased from Shanghai Aladdin Biochemical Technology Co., Ltd. (Shanghai, China). Single-walled carbon nanotubes (SWCNTs), chitosan, potassium ferricyanide, potassium ferrocyanide, potassium chloride, tetrachloroauric acid, BSA, glycerol, and potassium dihydrogen phosphate were purchased from Shanghai McLean Biochemical Technology Co., Ltd. (Shanghai, China). 4-(2-hydroxyethyl)-1-piperazineethanesulfonic acid (HEPES) buffer, yeast extract, peptone, nonyl phenoxypolyethoxylethanol (NP-40), a BCA protein assay kit, and phenylmethanesulfonyl fluoride (PMSF) were purchased from Sangon Biotech (Shanghai, China) Co., Ltd. (Shanghai, China). 2-Octanol (99.5%, chromatographic-grade) and normal paraffins (C_7_-C_22_) were purchased from Sigma-Aldrich (St. Louis, MO, USA and Shanghai, China). Titanium carbide (Ti_3_C_2_T_x_) MXene was purchased from Nanjing Xianfeng Nanotechnology Co., Ltd. (Nanjing, China). Millex-GP sterile filters (0.22 μm) were purchased from Merck (Darmstadt, Germany).

### 2.2. Malt Samples for Pyrazine Analysis

The following four malts were provided: munich malt (MM, moisture = 3.4%, EBC color = 16, extract yield = 81.5), pilsner malt (PM, moisture = 4.4%, EBC color = 3.7, extract yield = 82.5), crystal malt (CrM, moisture = 3.4%, EBC color = 328, extract yield = 75.9), and caramel malt (CaM, moisture = 2.7%, EBC color = 800, extract yield = 70.6).

### 2.3. Heterologous Expression of Olfactory Receptors

Yeast strain DJO1 (BY4741 trp1Δ::natMX) was transformed using the lithium acetate method [34]. Yeast colonies were grown in yeast extract peptone dextrose (YPD) medium at 30 °C for 48–72 h to induce OR5K1 expression.

### 2.4. Preparation of Nanovesicles (NVs)

The NVs were prepared according to previous experiments [35,36]. Yeast cell culture solution (OD_600_: 0.6–0.8) was dispensed into 50 mL centrifuge tubes and centrifuged at 5000 rpm for 2 min to collect yeast cells. Subsequently, 5 mL of ice water (0 °C) was added to the tube, mixed thoroughly, and centrifuged at 5000 rpm for 2 min to wash yeast cells twice. After that, the yeast cells were washed with 500 μL of cold lysis buffer solution (pH 6.8, 40 mM HEPES, 0.2% (*w/w*) NP-40, 10 mM DTT, 1 mM PMSF), collected by centrifugation at 5000 rpm for 2 min, resuspended in 1.5 mL of lysis buffer solution, and transferred into 1.5 mL centrifuge tubes. The NVs were prepared by the glass bead crushing method with 0.5 mm sterile glass beads. The yeast cell suspension was treated in a rapid grinder at 65 Hz for 1 min and then left on ice for 1 min. This process was cycled 8–10 times. The mixture was centrifuged at 6000 rpm and 4 °C for 24 min to remove unbroken cells and cell walls. The bottom layer of a nanovesicle suspension was obtained by centrifuging the supernatant at 4 °C for 60 min at 100,000 g. The nanovesicle suspension was resuspended in a lysis buffer solution with 10% (*w/w*) glycerol (the solvent was water), concentrated in the membrane fraction, and then stored at −80 °C for backup. The membrane fraction can be prepared in large quantities, frozen, and stored for several weeks without loss of biological activity before use. The protein concentration of the membrane preparation was determined using the BCA protein assay kit (Pierce, Brebières, France) with bovine serum albumin (BSA) as the standard. Before use, the nanovesicle suspension was diluted to a certain concentration in 10 mM PBS (pH 7.2–7.4), sonicated in ice-cold water for 10 min, and filtered through a sterile low-protein-binding filter (0.22 μm).

### 2.5. Western Blot

The expression of OR5K1 was verified using Western blot. NVs and yeast cell samples with 10 μg/mL protein content were electrophoresed on polyacrylamide gels to separate the target protein. Subsequently, the target proteins and markers in the gel were transferred onto a PVDF membrane for visualization. After that, the membranes were blocked in TBST containing 1% skimmed milk for 20 min and then incubated with anti-c-Myc tag mouse monoclonal antibody (Abbkine, Wuhan, China, diluted 1:1000 in TBST with 1% skimmed milk) for 1 h at 25 °C, followed by incubation with goat anti-mouse IgG secondary antibody (HRP-conjugated, Abbkine, Wuhan, China, diluted 1:5000 in TBST with 1% skimmed milk) for 1 h at 25 °C. Finally, the membrane was washed three times with TBST for 10 min each time. Eventually, the membranes were placed on plastic membranes and immersed in the color development solution and the blots were observed using a chemiluminescent developer.

### 2.6. Preparation of Nanomaterials

#### 2.6.1. Synthesis of ZIF-8@SWCNT

**Figure 1:**
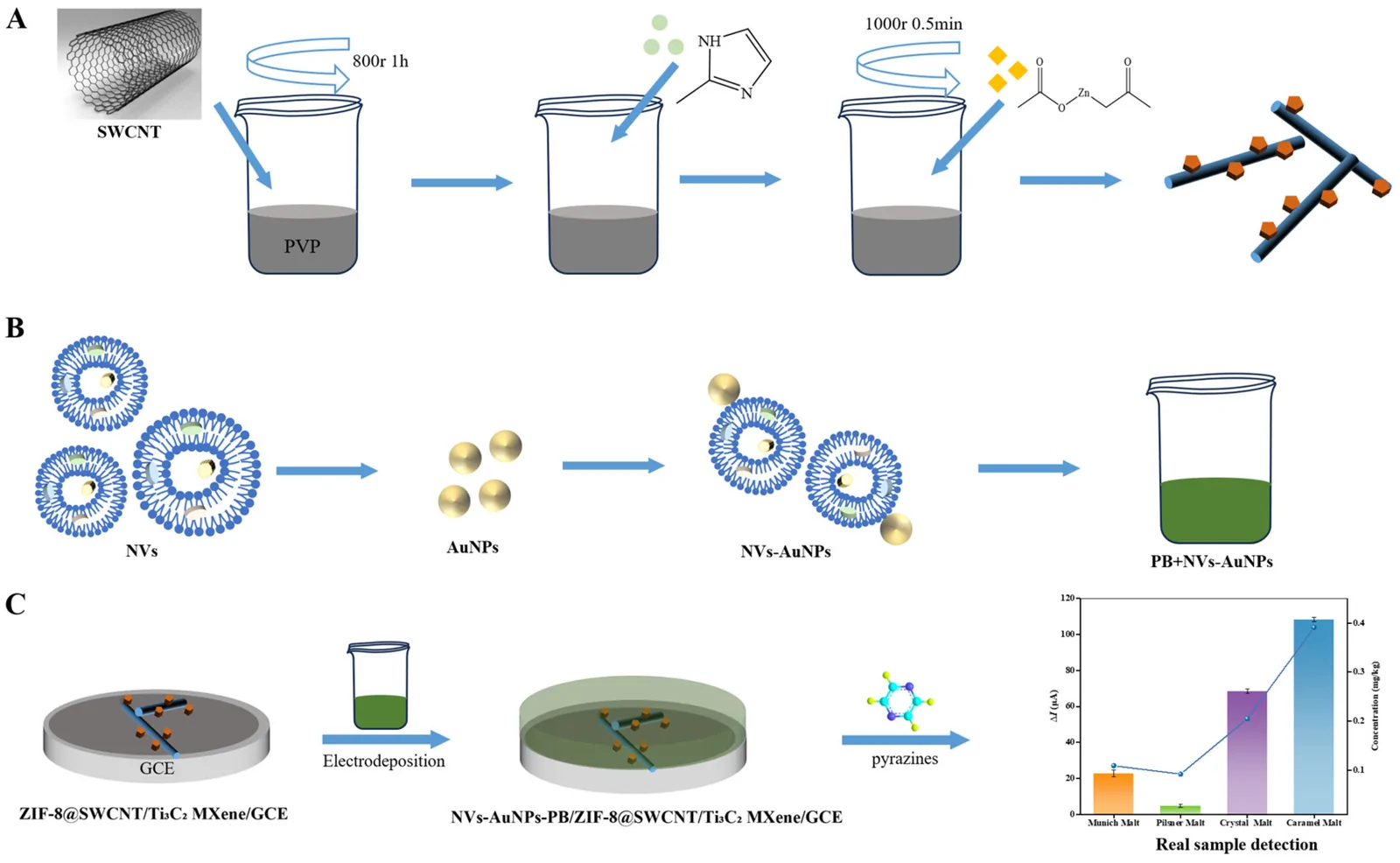
Schematic illustration of an olfactory biosensor based on the NVs-AuNPs-PB/ZIF-8@SWCNT/Ti_3_C_2_ MXene for the detection of pyrazine compounds. (**A**) Illustration of ZIF-8@SWCNT synthesis. (**B**,**C**) Schematic illustration of the fabrication process of an olfactory biosensor.

In total, 3 mg of SWCNTs were added to 2 mL of PVPP solution (5 mg/mL). The mixture was sonicated for 30 min and stirred at 800 rpm for 1 h. Subsequently, 2-methylimidazole with different masses (6, 12, 18, 24, and 30 mg) was separately added to the prepared SWCNT solution and sonicated for 30 min. After that, 2 mL of methanol solutions of zinc nitrate with different masses (11, 22, 33, 44, and 55 mg) were individually added to the dispersions, respectively. The mixture was stirred at 1000 rpm for 5 min and then allowed to stand at 25 °C for 24 h to prepare ZIF-8@SWCNT composites with different mass ratios (2:1, 4:1, 6:1, 8:1, and 10:1). PVPP plays two primary roles in the synthesis of ZIF-8@SWCNT. On the one hand, the polar groups of PVPP exhibit strong hydrophobic interactions with 2-methylimidazole molecules, enabling the adsorption of a large number of 2-methylimidazole groups. On the other hand, PVPP can improve the dispersibility of SWCNTs. These properties enable SWCNTs to generate more nucleation sites and promote the crystal nucleation of ZIF-8 on the surface of SWCNTs [37]. The obtained ZIF-8@SWCNT (Figure 1A) composites were washed with methanol several times, collected by centrifugation, and dried in a vacuum drying oven at 60 °C.

In total, 1.5 mg of ZIF-8@SWCNT powder was dissolved in 1 mL of 0.2% chitosan solution (containing 1% acetic acid). The mixture was sonicated for 4 h and then shaken for 2 h until a homogeneous and stable solution was obtained, which was stored at 4 °C.

#### 2.6.2. Preparation of AuNPs and NV–AuNP Complexes

AuNPs were prepared by the sodium citrate reduction method. First, 1 mL of 1% HAuCl_4_ solution was added to 100 mL of boiling ultrapure water with vigorous stirring. Then, 2.5 mL of 1% sodium citrate solution was rapidly added to the above solution. When the color of the solution changed from light gray to a distinct purple-red color, the mixture was continuously stirred and kept boiling for 10 min. The heating device was then removed, and the solution was cooled to 25 °C to obtain AuNPs (Figure 1B). Next, 4 mL of PVPP solution (25 mg/mL, MW = 55,000) was added dropwise to 30 mL of the prepared AuNPs, followed by stirring for 24 h. The mixture was centrifuged at 23,008× *g* for 30 min to collect stabilized AuNPs, which were then washed with methanol three times and finally dispersed in 2 mL of methanol.

Subsequently, 600 μL of AuNP solution and 400 μL of NV solution were mixed and shaken for 1 h in a shaker at 4 °C. The sulfhydryl (-SH) groups required for the formation of Au–S covalent bonds were derived from the naturally occurring thiol groups of membrane proteins (e.g., cysteine residues) present on the surface of the yeast-derived NVs, which did not require additional chemical thiolation. Then the mixture was left at 4 °C for 1 h so that stable NV–AuNP complexes were fully formed by Au-S covalent bonds between the sulfhydryl groups on the NVs and AuNPs, which were stored at 4 °C.

#### 2.6.3. Preparation of Electrodeposition Solution

In total, 0.125 g FeCl_3_, 0.1644 g K_3_[Fe(CN)_6_] ^3−/4−^, and 1.491 g KCl were added sequentially to 200 mL of 0.05% chitosan solution (containing 1% acetic acid solution). Afterward, the mixture was stirred at 25 °C overnight to obtain a dark green Prussian blue solution (PB). An electrodeposition solution was obtained by adding 300 μL of NV–AuNP solution to 6.7 mL of PB solution (Figure 1B).

### 2.7. Fabrication of Olfactory Biosensors

The glassy carbon electrodes (GCEs) were polished sequentially with 0.3 μm, 0.1 μm, and 0.05 μm aluminum powder on a polishing cloth. Subsequently, the GCEs were ultrasonicated in ultrapure water and ethanol for 30 s each and then blown dry with nitrogen gas. All electrochemical tests in this work were conducted on a CHI 660E electrochemical workstation (Chenhua Instrument Co., Ltd., Shanghai, China) in a 5 mM [Fe(CN)_6_] solution containing 0.1 M KCl. As shown in Figure 1C, the biosensor was constructed as follows: 8 μL of Ti_3_C_2_ MXene dispersion (0.25 mg/L, diluted in 0.05% chitosan solution) was dropped onto the electrode surface and dried at room temperature. Then, 8 μL of the prepared ZIF-8@SWCNT solution was added to the electrode surface, which was then dried at room temperature. Afterwards, the modified electrode was immersed in the electrodeposition solution, and the NV–AuNP–PB solution was electrodeposited onto the above electrode surface using cyclic voltammetry (CV). The CV parameters were set as follows: scan ranging from −0.2 V to 0.6 V, scan rate of 100 mV/s, and scan number of 15 cycles. The DPV measurements were set as follows: scan ranging from −0.2 V to 0.6 V, potential increment of 0.004 V, pulse amplitude of 0.05 V (50 mV), and pulse width of 0.05 s (50 ms). After that, the electrode surface was gently rinsed with ultrapure water to remove the undeposited electrodeposited solution and dried naturally at room temperature. Finally, 6 μL of 0.5% BSA solution was applied to the surface to block non-specific binding sites, followed by natural air-drying at room temperature for subsequent use. All electrochemical measurements in this study were performed in triplicate to ensure experimental repeatability and data reliability.

### 2.8. Gas Chromatography–Time-of-Flight Mass Spectrometry Analysis (GC-ToF/MS)

#### 2.8.1. Sample Pretreatment

An appropriate amount of malt samples was placed in a grinder and ground for 30–60 s at a rotation speed of 10,000–12,000 rpm. After grinding, the sample was sieved, and the resulting powder was collected in a dry and clean beaker. Exactly 10 g of the malt powder was weighed and transferred into a 250 mL stoppered Erlenmeyer flask. The volume of 95% ethanol required was calculated according to a solid-to-liquid ratio of 1:5 (g:mL) and slowly added to the flask. The mixture was vortexed for 30 s to fully disperse the malt powder in ethanol and avoid agglomeration. Extraction was performed at 70 °C for 2 h, and this extraction process was repeated twice. The combined extracts were concentrated under reduced pressure (45 °C, −0.08 to −0.09 MPa) to obtain the malt extract, which was hermetically sealed and stored at −80 °C. The extraction conditions (solid-to-liquid ratio of 1:5, 95% ethanol, 70 °C, 2 h) were optimized based on preliminary experiments to ensure maximum recovery of pyrazine compounds from the malt matrix. The extraction efficiency was confirmed by performing the extraction twice and combining the extracts, and the repeatability was ensured by analyzing all samples in triplicate.

#### 2.8.2. GC-TOF/MS Analysis Conditions

Initially, malt extract sample (5 g) and sodium chloride (1 g) were added and vortexed until completely dissolved. Subsequently, 50 μL of 2-octanol (100 mg/L, internal standard) and 10 g of anhydrous sodium sulfate were added. The mixture was allowed to stand at 4 °C and then filtered. The analysis of extracted volatile organic compounds (VOCs) was conducted using a GC-ToF/MS (gas chromatography–time-of-flight mass spectrometry) system with an Agilent 7890A gas chromatograph (Agilent Technologies, Inc., Santa Clara, CA, USA) equipped with a DB-Wax capillary column (30 m × 0.25 mm × 0.25 μm). Each sample was analyzed in triplicate. The inlet temperature was set to 250 °C, and helium (99.999%) was used as the carrier gas with a column flow of 2 mL/min and a split ratio of 5:1. The oven temperature was initially held at 50 °C and then increased to 80 °C at a rate of 5 °C/min with no hold. It was further increased to 90 °C at 2 °C/min and then increased to 180 °C at 10 °C/min. It was finally increased to 220 °C at 2 °C/min, where it was maintained for 5 min. The mass spectrometer was operated in electron ionization mode, with an electron energy of 70 eV, an ion source temperature of 200 °C, a transmission line temperature of 260 °C, and a full scan range of 29–450 *m/z*. The RI was calculated under the same chromatographic conditions using a series of alkanes (C_7_–C_22_) following the standard linear retention-index calculation method described in the literature [38]. Semi-quantitative analysis was performed using the internal standard method. The quantitative results were expressed as the mean ± standard deviation.

### 2.9. Preparation of Structures of Olfactory Receptor and Pyrazine Odor Molecules

The 3D structure of the human olfactory receptor OR5K1 (Uniprot ID: Q8NHB7) was selected to investigate its interactions with pyrazine odorant molecules. The structure of the human olfactory receptor was constructed by AlphaFold 3. The optimal model was downloaded and saved in protein data bank (PDB) file format. The PROCHECK Ramachandran plot for evaluating the structural quality and rationality of the olfactory receptor protein was obtained from the online tool SAVES: https://saves.mbi.ucla.edu/ (accessed on 10 September 2024).

The structures of six pyrazine compounds were drawn using ChemDraw Version 22.0 software, and their names are listed in Table S1. The small molecule format (SDF) files of the aroma compounds were retrieved from the PubChem database: https://pubchem.ncbi.nlm.nih.gov/ (accessed on 8 September 2024), which were then converted from SDF format to PDB format using OpenBabel Version 2.4.1 software and saved. Subsequently, the structural energy minimization of these pyrazine compounds was performed by the MM2 protocol implemented in Chem3D Version 22.0 software.

### 2.10. Molecular Docking

AutoDock (Version 4.2, Scripps Research, San Diego, CA, USA) was employed for the preprocessing of protein and ligand structures. The olfactory receptor protein was pretreated by removing water molecules, adding hydrogen atoms, and then converting it into a PDBQT format file. The ligands were subjected to hydrogenation and charge assignment, followed by torsion tree setup. Finally, the processed ligands were saved as PDBQT format files. Based on the predicted binding pocket, molecular docking was performed to generate the most rational conformation. The ligand and OR conformation files were imported into PyMOL 3.1.8 software (DeLano Scientific LLC, South San Francisco, CA, USA) to obtain binding site interaction diagrams. LigPlot^+^ (Version 2.2) software (EMBL-EBI, Cambridge, UK) was used to generate 2D interaction diagrams and analyze the interaction mechanism between the olfactory receptor OR5K1 and pyrazine odorant molecules.

### 2.11. In Silico Site-Directed Mutagenesis Simulation

In silico site-directed mutagenesis simulation was performed using PyMOL software (DeLano Scientific LLC, South San Francisco, CA, USA). Key amino acid residues in the olfactory receptor OR5K1 were individually substituted with alanine or glycine by the mutagenesis tool in the wizard toolbar [39]. Specifically, the “Mutagenesis” option was clicked, followed by selection of the protein target. Under the “No Mutation” dropdown menu, alanine or glycine was chosen, then the “Apply” and “Finish” buttons were clicked sequentially. The 3D structure of the mutated protein was saved as a PDB file. The mutated olfactory receptor protein was pretreated by removing water molecules, adding hydrogen atoms, and subsequently converting it into a PDBQT format file. Semi-flexible molecular docking was employed to dock the ligands (in PDBQT format) into the mutated olfactory receptor protein.

### 2.12. Statistical Analysis

GC-ToF/MS data were processed and analyzed via Chroma TOF Software (v5.50.55.0, LECO Corporation, St. Joseph, MI, USA) for the Pegasus BT system. The characterization of electrochemical behaviors of olfactory biosensor graphs was created using Origin 2021 software (OriginLab Corporation, Northampton, MA, USA). Group differences were analyzed using Student’s *t*-test and one-way ANOVA with SPSS 27.0 (IBM Corporation, Armonk, NY, USA). The marked areas with letters denote *p* < 0.05 compared with each control group. All results were expressed as the mean ± standard deviation.

## 3. Results and Discussion

### 3.1. Characterization of Olfactory Biosensor Based on NV–AuNP–PB/ZIF-8@SWCNT/Ti_3_C_2_ MXene

#### 3.1.1. Characterization of the Olfactory Receptor

**Figure 2:**
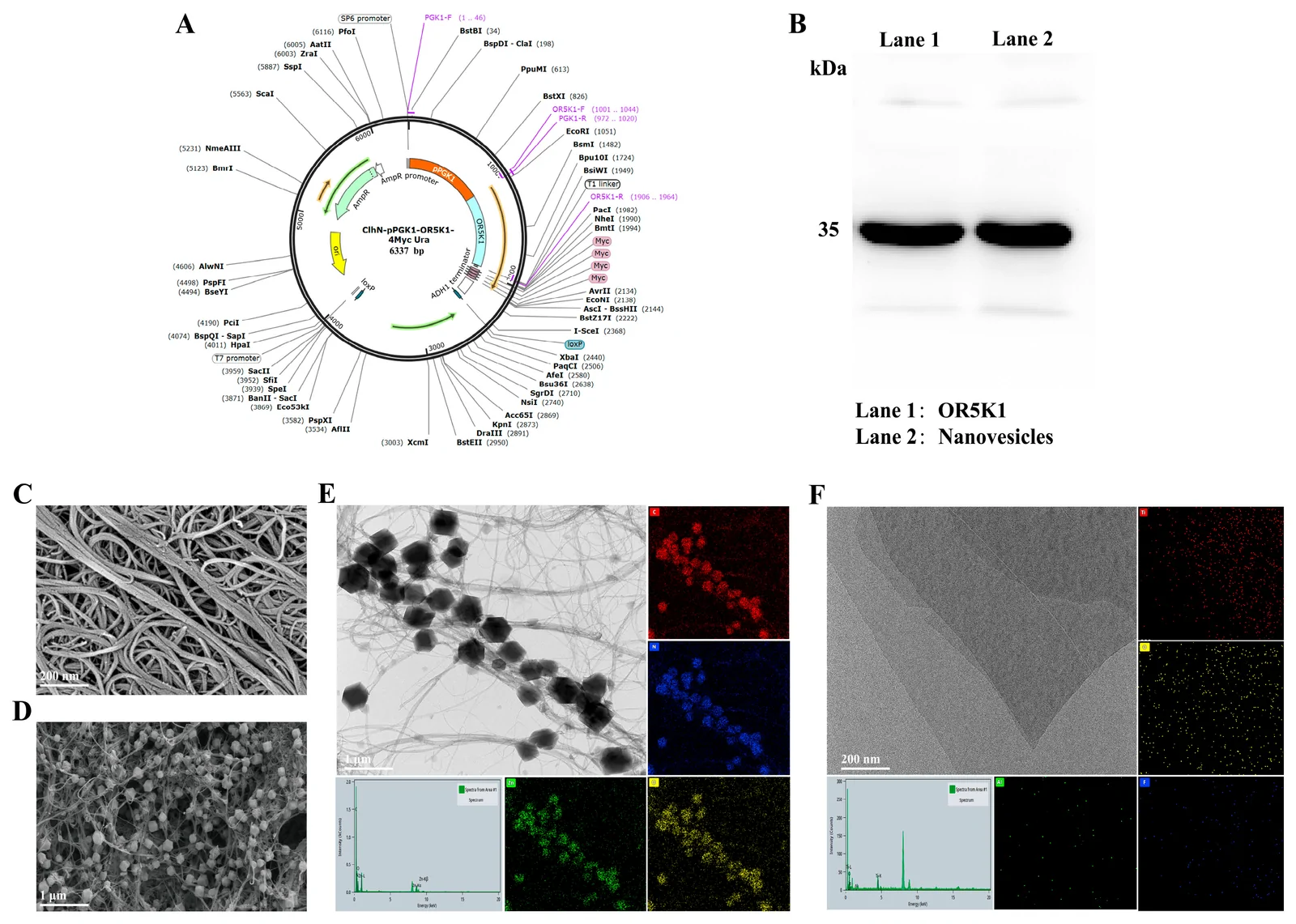
Characterization of the OR5K1 produced in yeast DJO1 and the nanomaterials. (**A**) The amino acid sequence of plasmid ClhN-4Myc-Ura encoding OR5K1. (**B**) Western blot analysis of OR5K1 in yeast cells and cell-derived NVs. (**C**) SEM images of SWCNT. (**D**) SEM images ZIF-8@SWCNT. (**E**) TEM images and EDS mapping of ZIF-8@SWCNT. (**F**) TEM images and EDS mapping of Ti_3_C_2_ MXene.

To verify whether the human olfactory receptor OR5K1 was successfully heterologously expressed in *Saccharomyces cerevisiae* DJ01, Western blot analysis was performed to confirm the expression of OR5K1 in yeast cells and yeast-derived NVs (Figure 2B). The electrophoretic bands of cells and NVs are represented in lane 1 and lane 2, respectively. The black bands at 35 kDa correspond to the molecular weight of OR5K1, indicating the successful overexpression of OR5K1.

#### 3.1.2. Characterization of the Nanomaterials

To investigate the morphological and structural characteristics of the synthesized nanomaterials, this study employed scanning electron microscopy (SEM, Zeiss GeminiSEM 300, Oberkochen, Germany) and transmission electron microscopy (TEM, Thermo Scientific Talos F200X, Waltham, MA, USA) for characterization and analyzed the elemental distribution by energy-dispersive X-ray spectroscopy (EDS) mapping. For SEM imaging, the SWCNT and ZIF-8@SWCNT samples were prepared by drop-casting the ultrasonically dispersed dispersions onto silicon wafers and sputter-coated with a thin layer of gold to enhance conductivity and then placed in the SEM apparatus for imaging at an accelerating voltage of 5 kV with an emission current of 10 μA and a working distance of 8 mm. For TEM imaging, the ZIF-8@SWCNT and Ti_3_C_2_ MXene samples were prepared by depositing a drop of the dispersion onto a carbon-coated copper grid, rapidly dried at room temperature, and then placed in the TEM apparatus for imaging at an accelerating voltage of 200 kV with an emission current of 15 μA. Elemental mapping and compositional analysis were performed using EDS attached to the TEM system, operated at an accelerating voltage of 200 kV with an acquisition time of 120 s.

SEM was used to observe SWCNT and ZIF-8@SWCNTs, while TEM was applied to characterize the morphology and structure of ZIF-8@SWCNT and Ti_3_C_2_ MXene. MOFs have emerged as ideal carriers for sensor construction due to their ordered crystal structures, tunable functional groups, and high specific surface areas [40,41]. SEM image of SWCNT (Figure 2C) shows that the carbon nanotubes exhibited an elongated tubular structure. ZIF-8 is a non-toxic material synthesized from zinc ions and 2-methylimidazoline, serving as an ideal carrier. It not only enhances protein loading capacity but also improves the stability and activity of immobilized biomolecules [42]. ZIF-8’s insufficient conductivity restricts electrochemical signal transduction [43]. In this regard, SWCNTs and Ti_3_C_2_ MXene are promising candidates for incorporation into ZIF-8 [23,24]. SWCNTs possess a large specific surface area that significantly improves electron transport efficiency [44]. The SEM image of ZIF-8@SWCNTs (Figure 2D) revealed that ZIF-8 particles were penetrated and connected in series by SWCNTs, with a rough and porous surface. The ZIF-8 particles presented an irregular shape, and their particle size ranged from 330 to 550 nm. TEM images of ZIF-8@SWCNT (Figure 2D) exhibit a necklace-like morphology, indicating the successful in situ growth of ZIF-8 nanocrystals on carbon nanotube fibers by heterogeneous nucleation. The ZIF-8 particles were densely and uniformly encapsulated on the surface of carbon nanotubes and appeared spherical, which was consistent with the results of the SEM image. The EDS mapping showed a uniform distribution of Zn, O, C, and N elements throughout the fibrous skeleton, and the C-spectra correlated well with the TEM image of the carbon nanotubes. In the ZIF-8@SWCNT structure, ZIF-8 possessed a large specific surface area, while SWCNTs provided an electron transfer pathway, thereby improving the electrical conductivity of ZIF-8. Their synergistic effect was conducive to enhancing the electrochemical performance of the electrode material.

As a type of two-dimensional transition metal carbide material, Ti_3_C_2_ MXene exhibits metallic conductivity and hydrophilicity due to its surface functional groups, thereby amplifying the electrochemical sensing signals of sensors. The rationale for selecting Ti_3_C_2_ MXene in this study lies in its unique combination of properties: its metallic conductivity facilitates efficient electron transfer, its hydrophilic surface enables good dispersion in aqueous solution and compatibility with biological components, and its layered structure provides a large surface area for the deposition of other nanomaterials and the immobilization of biomolecules. The TEM image of Ti_3_C_2_ MXene visualized the layered structure of the nanosheets after etching. As shown in Figure 2F, the presence of multiple visible nanosheets indicated that MXene had been effectively etched from its MAX phase. The resulting nanosheets typically exhibited a very thin, multilayered structure with sharp, distinct edges, transparent lamellae, and a smooth surface, as observed in the image. They possessed negligible nanoscale defects and a certain degree of flexibility, with folding and stacking phenomena observed in some regions, all of which are typical characteristics of two-dimensional nanomaterials. In addition, the EDS mapping revealed that the MXene nanosheets were obtained by the selective etching of the Al layer from the Ti_3_AlC_2_ MAX phase using LiF/HCl, followed by ultrasonic vibration in aqueous solution and exfoliation under the protection of flowing argon [45]. No obvious particulate impurities or characteristic signals of other phases were observed in the image, suggesting high sample purity. The EDS mapping results further confirmed the high purity, which showed a high content of Ti and O elements but an extremely low content of F and Al elements. In conclusion, both SEM and TEM results indicated the successful synthesis of ZIF-8@MWCNTs and Ti_3_C_2_ MXene composites.

### 3.2. Characterization of Electrochemical Behaviors of the Olfactory Biosensor

**Figure 3:**
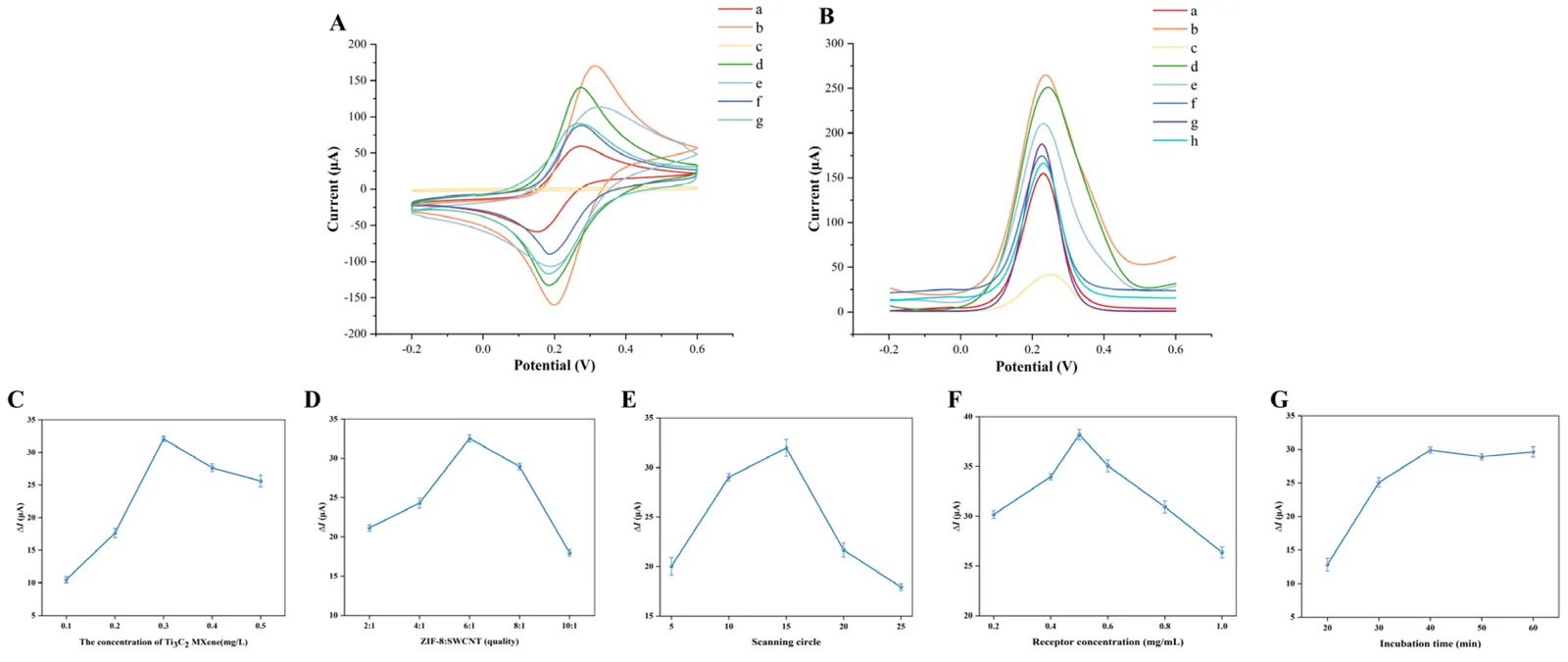
Characterization of the electrochemical behaviors of the olfactory biosensor and optimization of experimental conditions. (**A**) CV. (**B**) DPV curves of different modified electrodes in solution of 5.0 mM [Fe(CN)_6_]^3−/4−^ containing 0.1 M KCl, (a) GCE, (b) Ti_3_C_2_ MXene, (c) ZIF-8@SWCNT/GCE, (d) ZIF-8@SWCNT/Ti_3_C_2_ MXene/GCE, (e) AuNP–PB/ZIF-8@SWCNT/Ti_3_C_2_ MXene/GCE, (f) OR5K1–NV–AuNP–PB/ZIF-8@SWCNT/Ti_3_C_2_ MXene/GCE, (g) BSA/OR5K1–NV–AuNP–PB/ZIF-8@SWCNT/Ti_3_C_2_ MXene/GCE, and (h) BSA/OR5K1–NV–AuNP–PB/ZIF-8@SWCNT/Ti_3_C_2_ MXene-TMP. (**C**) The concentration of Ti_3_C_2_ MXene. (**D**) The mass ratio of ZIF-8 to SWCNT. (**E**) Scanning circle. (**F**) Receptor concentration. (**G**) Incubation time.

To characterize the sensor fabrication process, CV and differential pulse voltammetry (DPV) were employed. As shown in Figure 3A a, b, after modification of the electrode with Ti_3_C_2_ MXene, the peak current increased significantly, which was attributed to the excellent electrical conductivity of Ti_3_C_2_ MXene. After ZIF-8@SWCNTs were modified on the electrode, the peak current decreased sharply due to the weak conductivity of ZIF-8 (Figure 3A a–c). When ZIF-8@SWCNTs/Ti_3_C_2_ MXene was modified on the electrode, a distinct increase in current was observed owing to the conductivity of Ti_3_C_2_ MXene (Figure 3A c–d). After AuNP–PB was electrodeposited on the surface of the electrode, the AuNP–PB/ZIF-8@SWCNT/Ti_3_C_2_ MXene-modified electrode exhibited a relatively high peak current value, which was because the synergistic effect of AuNPs and PB significantly facilitated electron transfer. PB serves two critical functions in the biosensor construction. First, PB acts as an electron transfer mediator. The redox probe [Fe(CN)_6_]^3−^/^4−^ is used in the electrolyte solution to monitor the electrochemical response of the biosensor. PB, which itself is an iron-based mixed-valence compound, facilitates electron transfer between the electrode and the redox probe by providing an electrocatalytic pathway, thereby increasing the peak current response. This signal amplification effect is essential for achieving the low detection limit (10^−14^ M) of the sensor. Second, PB serves as a matrix for receptor immobilization. The co-deposition of PB with AuNPs and NVs creates a porous and biocompatible film that not only maintains the biological activity of OR5K1 but also provides a stable microenvironment for the receptor, contributing to the long-term stability of the biosensor. The roles of PB and AuNPs are further supported by the stepwise electrode modification data presented in Figure 3A,B. The significant increase in peak current from the ZIF-8@SWCNT/Ti_3_C_2_ MXene/GCE (curve d) to the AuNP–PB/ZIF-8@SWCNT/Ti_3_C_2_ MXene/GCE (curve e) confirms the synergistic signal amplification effect of AuNPs and PB, which facilitate electron transfer at the electrode interface. The subsequent decrease in peak current upon modification with NV–AuNP–PB (curve f) confirms the successful immobilization of the receptor-containing NVs. However, modification of the electrode with NV–AuNP–PB decreased the peak current, confirming successful immobilization of the NVs. After that, the biosensor was incubated with BSA to block the non-specific binding sites, and a decrease in peak current was observed. When the BSA/NV–AuNP–PB/ZIF-8@SWCNT/Ti_3_C_2_ MXene/GCE was incubated with TMP, the peak current value decreased further. This was due to the blocking effect of the formed receptor–TMP complex. These results confirmed the successful construction of an olfactory biosensor.

After that, the biosensor was incubated with BSA to block the non-specific binding sites, and a decrease in peak current was observed. When the BSA/NV–AuNP–PB/ZIF-8@SWCNT/Ti_3_C_2_ MXene/GCE was incubated with TMP, the peak current value decreased further. This was due to the blocking effect of the formed receptor–TMP complex. These results confirmed the successful construction of an olfactory biosensor.

### 3.3. Optimization of Conditions for Pyrazine Detection

To enhance the electrochemical performance of olfactory biosensors, five key factors were optimized, including the concentration of Ti_3_C_2_ MXene, the mass ratio of ZIF-8@SWCNT, the number of scanning cycles of the electrodeposition solution, the concentration of the NVs, and the incubation time.

Ti_3_C_2_ MXene as a substrate material exerts a significant impact on the sensitivity of the biosensor and its coupling performance with other nanomaterials. Figure 3C shows that the current response values increase with the increase in Ti_3_C_2_ MXene concentration, reaching a maximum value at 0.3 mg/L, and then decrease slowly. This is because a proper density distribution of Ti_3_C_2_ MXene on the electrode can significantly enhance the conductivity of the material. A low concentration tends to hinder electron transfer, whereas an excessively high concentration of Ti_3_C_2_ MXene will cause stacking on the electrode with a fixed surface area, which is unfavorable for electron transfer. Therefore, the optimal concentration of Ti_3_C_2_ MXene is determined to be 0.3 mg/L. The composite of ZIF-8 and SWCNT has a great influence on the electrochemical activity of the electrode surface and the loading capacity of the NVs. As shown in Figure 3D, the current response reaches the maximum value when the mass ratio of ZIF-8 to SWCNTs is 6:1. This is probably because the increased proportion of ZIF-8 provided a larger specific surface area for the attachment of the NVs, thereby increasing the amount of nanovesicle immobilization and reducing the steric hindrance between NVs. When a high proportion of ZIF-8 was grown on the SWCNT, the stacked ZIF-8 reduced the sensitivity of the biosensor owing to its poor conductivity. Thus, the optimal mass ratio of ZIF-8 to SWCNTs is confirmed as 6:1. The amount of immobilized NVs is governed by the PB electrodeposition scanning cycles and the nanovesicle concentration. As shown in Figure 3E, the current difference increases with the increase in scanning cycles and peaks at 15 cycles. This is because the layer-by-layer stacking of the Prussian blue deposition film increases the electrochemically active surface area of the electrode and the quantity of NVs. Nevertheless, an overly thick PB film and a high concentration of NVs can lead to excessive accumulation, thereby decreasing the response current (Figure 3E,F).

Therefore, the optimal scanning cycles and receptor concentration are 15 cycles and 0.5 mg/mL, respectively. Incubation time is crucial for the stability of the biosensor. The incubation time of pyrazine compounds on the OR5K1 nanovesicle-modified electrode was also investigated. As shown in Figure 3G, the current variation reaches the maximum value at an incubation time of 40 min and then remains stable. This is because the binding between NVs and pyrazine molecules requires a certain reaction time to form a stable receptor–pyrazine complex. Therefore, the optimal incubation time is determined to be 40 min.

In summary, the optimal conditions were as follows: the concentration of Ti_3_C_2_ MXene was 0.3 mg/mL, the mass ratio of ZIF-8 to SWCNTs was 6:1, there were 15 scanning cycles of the electrodeposition solution, the concentration of NVs was 0.5 mg/mL, and the incubation time was 40 min.

### 3.4. Analytical Performance for Pyrazine Compound Detection

**Figure 4:**
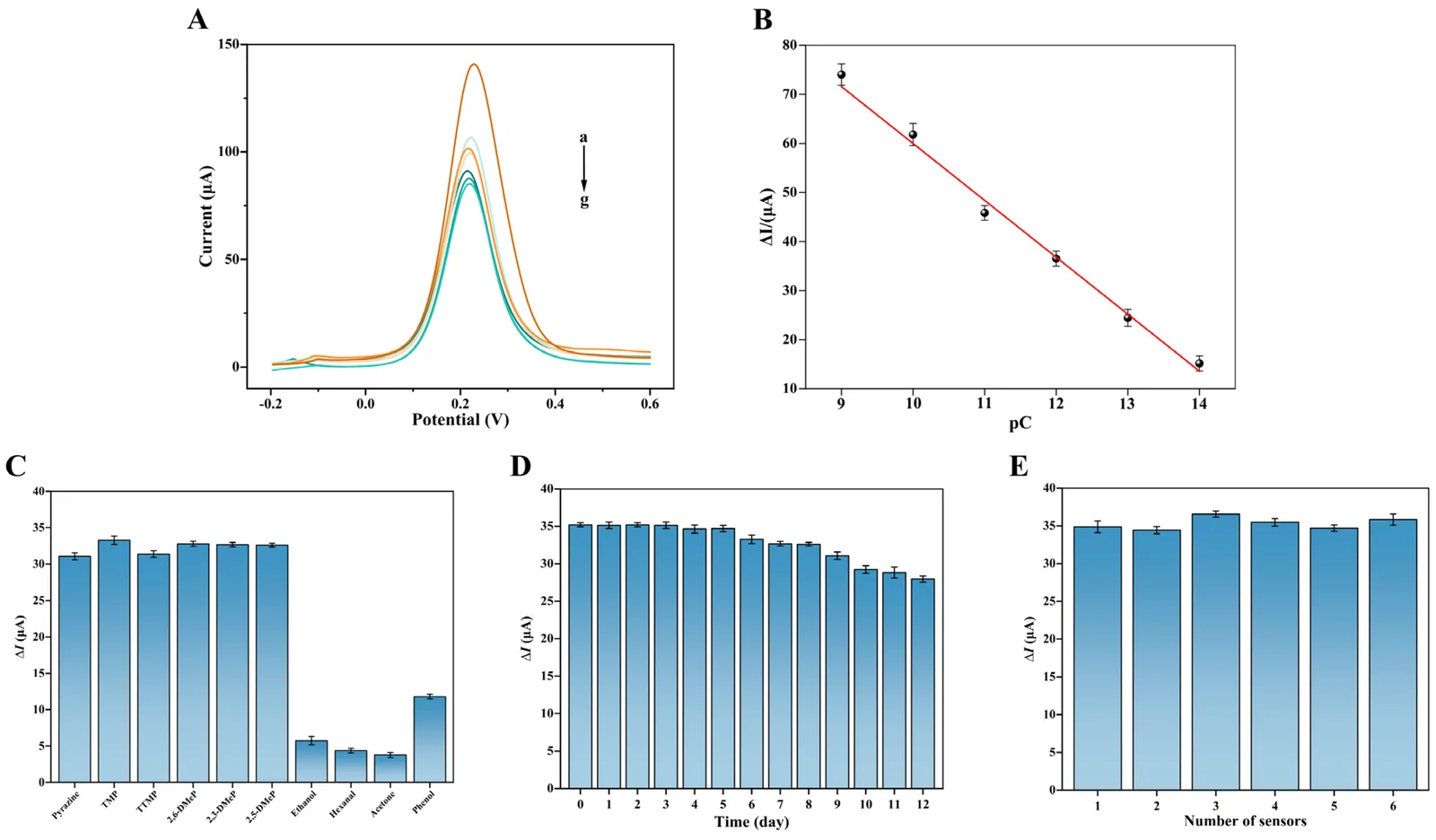
Response of olfactory biosensor to pyrazine compounds. (**A**) DPV response of OR5K1–NV–AuNP–PB/ZIF-8@SWCNT/Ti_3_C_2_ MXene/GCE toward various concentrations of TMP (a–g: 0, 10 ^−14^, 10^−13^, 10^−12^, 10^−11^, 10^−10^, and 10^−9^ M). (**B**) Calibration curve of OR5K1–NV–AuNP–PB/ZIF-8@SWCNT/Ti_3_C_2_ MXene/GCE for the detection of TMP (*n* = 5). (**C**) Selective response of the sensor to pyrazine compounds (pyrazine, TMP, TTMP, 2,6-DMeP, 2,3-DMeP, 2,5-DMeP, ethanol, hexanal, acetone, phenol). (**D**) Sensor responses to TMP during storage. (**E**) Reproducibility of sensor responses.

Under the optimal conditions, DPV was used to explore the dependence of the olfactory biosensor response on the concentration of TMP within the 5 mM [Fe(CN)_6_]^3−/4−^ solution containing 0.1 M KCl. TMP was selected as a representative pyrazine standard for constructing the calibration curve, based on preliminary data showing that it elicited the highest current response among the tested pyrazine compounds. Figure 4A shows that the reduction peak current (ΔI) of DPV gradually decreased as the concentration of 2,3,5-trimethylpyrazine increased from 10^−14^ M to 10^−9^ M, indicating that the olfactory biosensor exhibited excellent electrochemical performance. As presented in Figure 4B, it could be observed that the current response difference (ΔI) increased with the rise in TMP concentration. Figure 4C shows that the current response of the biosensor exhibited a good linear relationship with TMP in the concentration range of 10^−14^ to 10^−9^ M. The calibration equation was determined as ΔI (μA) = (−11.60 ± 0.55) pC + (164.4 ± 6.0), with a correlation coefficient of 0.9889. The limit of detection was defined as the lowest analyte concentration that could be reliably distinguished from the blank signal at a specified confidence level and was calculated as 10^−14^ M using the 3σ/S criterion. Here, σ is the standard deviation of the DPV responses from ten independent blank measurements and S is the absolute slope of the calibration curve (|S| = 11.60 μA/pC). This value was consistent with the experimental verification at 10^−14^ M, confirming the high sensitivity of the OR5K1-based olfactory biosensor.

The excellent sensitivity of the olfactory biosensor was attributed to the dual signal amplification effect of ZIF-8@SWCNT/Ti_3_C_2_ MXene and AuNPs/PB. As a carrier, the ZIF-8@SWCNT/Ti_3_C_2_ MXene composite provided a large specific surface area for the attachment of the electrodeposition solution and the immobilization of NVs. In addition, PB exhibited excellent electrocatalytic activity and biocompatibility, and the PB-AuNP film formed by the co-deposition of PB and AuNPs could improve the electrochemical performance and sensitivity of the sensor [46]. Furthermore, the co-deposition of PB and receptors enabled the effective immobilization of receptors on the sensing interface, and the formed PB–AuNP–receptor film could better maintain receptor activity as a protective layer, thereby prolonging the service life of the sensor.

### 3.5. Selectivity, Stability, and Repeatability of Olfactory Biosensor

The selectivity, repeatability, and stability of olfactory biosensor were verified to assess its performance. Figure 4C shows the current responses of the biosensor to 10^−9^ M pyrazine, TMP, TTMP, 2,6-DMeP, 2,3-DMeP, and 2,5-DMeP as well as interfering substances, including ethanol, hexanal, acetone, and phenol (10^−7^ M). The biosensor exhibited significantly higher current variations toward pyrazine compounds than toward interfering substances, indicating that the olfactory receptor OR5K1 has excellent selectivity for pyrazine compounds. However, it was found that the olfactory receptor showed a relatively high current response to phenol compared with the other three interfering substances. It is hypothesized that this may be because phenol has a benzene ring structure similar to that of pyrazine compounds, which may trigger similar signal responses. In the current study, we evaluated the selectivity of the biosensor against a series of pyrazine analogs (pyrazine, TMP, TTMP, 2,6-DMeP, 2,3-DMeP, 2,5-DMeP), as well as typical food-related interferents (ethanol, hexanal, acetone, and phenol). In future work, we will expand the selectivity panel to include pyridines, pyrroles, and other heterocyclic analogs to further validate the specificity of the OR5K1-based biosensor.

The stability of the olfactory biosensor was tested by storing it in a refrigerator at 4 °C (Figure 4D). The current signal of the olfactory biosensor toward 10^−9^ M TMP showed a decreasing trend during storage. This is because, in the in vivo environment, olfactory receptors can maintain good structure and activity in olfactory tissues, whereas in the in vitro environment, an inappropriate physiological environment can negatively affect the stability of the hybrid system composed of receptors and bioelectronic devices in various ways [37]. Factors such as pH, temperature, or the presence of interfering substances can all affect the structural integrity and binding affinity of the receptors. For example, temperature changes can cause receptor denaturation or activity decrease, leading to decreased sensitivity or specificity. These factors jointly degrade the performance and reliability of bioelectronic devices. Nevertheless, the biosensor constructed in this study retained more than 79% of its initial signal after 12 days of storage, which may be attributed to the protective effect of the phospholipid bilayer and PB–AuNP film on the receptors. These results demonstrate that the olfactory biosensor has good stability and very good storage stability.

Another key factor to be considered in evaluating the biosensor is repeatability. Six groups of electrodes (five electrodes per group) fabricated under the same conditions were stored in a refrigerator at 4 °C, and no significant variation was observed in their response currents (*p* > 0.05), with a relative standard deviation (RSD) of 2.54%, indicating that the constructed biosensor has good repeatability (Figure 4E). However, since the olfactory receptor proteins are not in a hydrated environment, the interaction between the receptors and gas molecules is limited. Therefore, this biosensor is only applicable for the detection of pyrazine compounds in the liquid phase.

### 3.6. Genuine Sample Analysis

The key VOCs generated during malt roasting are nitrogen-containing, oxygen-containing, and sulfur-containing heterocyclic compounds. Nitrogen-containing heterocyclic compounds are products of the Maillard reaction, mainly including pyrazines, pyridines, and pyrroles. Vandecan et al. [47] found that among pyrazine compounds, 2-ethyl-3,5-dimethylpyrazine and TMP are key aroma-active compounds in caramel malt. Kim et al. [48] demonstrated that 2-methylpyrazine and 2,6-DMeP are important aroma substances in roasted barley, characterized by a mainly roasted odor. The four real malt samples (MM, PM, CrM, CaM) used in the experiment differ mainly in roasting degree. PM has the lightest roasting degree, presenting a light yellow color (EBC = 3.7) with a mild and pure flavor, dominated by basic malt aroma. MM undergoes light-to-medium roasting, showing a light amber color (EBC = 16) with both malt sweetness and a slight nutty/caramel aroma. CrM is processed by medium-to-deep roasting, exhibiting a deep amber color (EBC = 328) with a rich caramel and honey aroma. CaM has the highest roasting degree, appearing dark brown to nearly black (EBC = 800), with intense burnt and toffee notes accompanied by a slight bitterness.

**Figure 5:**
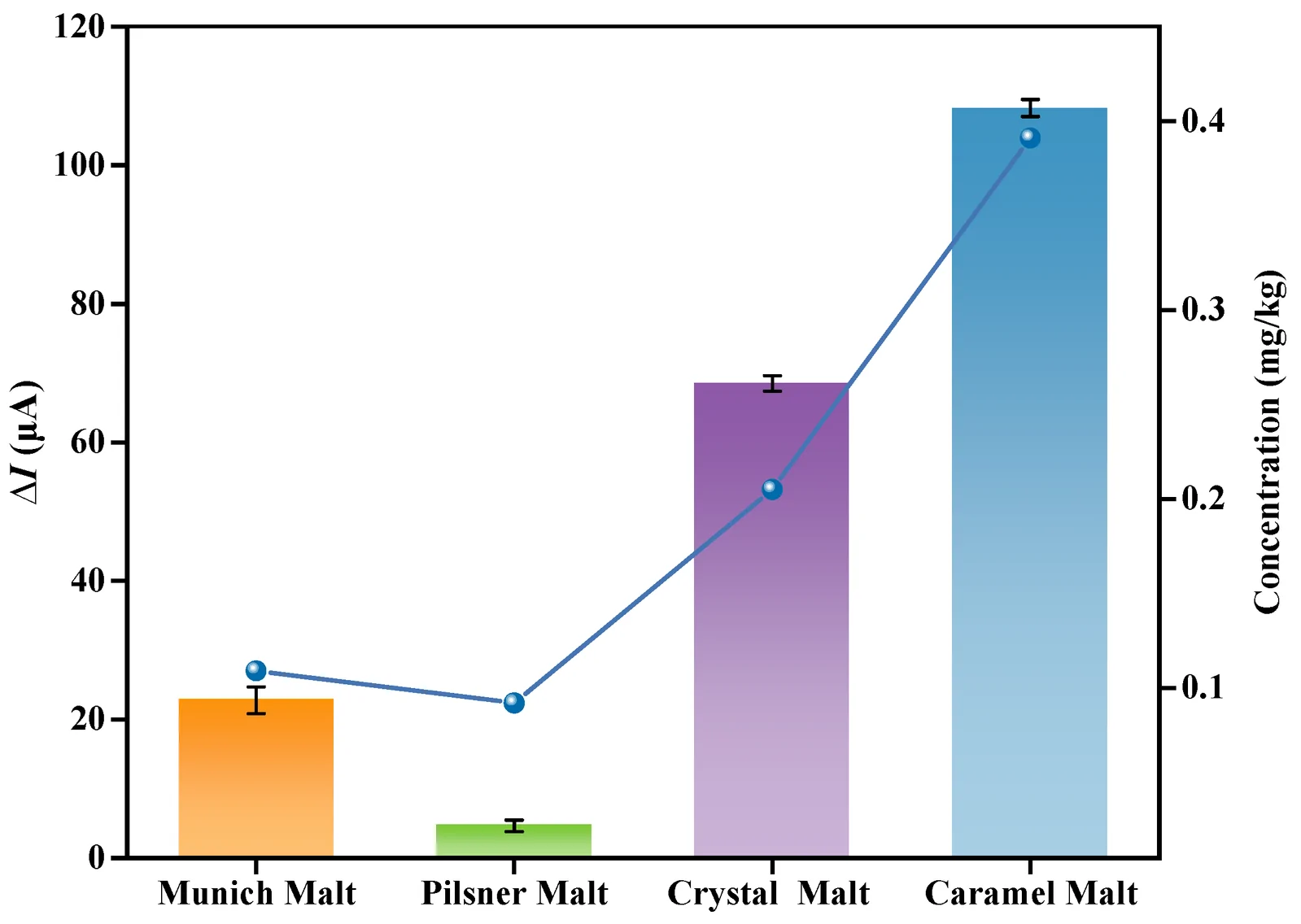
Detection of pyrazine compounds in four malt samples by olfactory biosensor and GC-TOF/MS. The bar chart represents the current response values of the olfactory sensor and the line graph represents the concentrations of pyrazine compounds in malt determined by GC-TOF/MS.

Table S2 presents the GC-TOF/MS detection results of the four real malt samples. A total of 211 VOCs were identified and categorized into alcohols, aldehydes, ketones, esters, acids, heterocyclic compounds, hydrocarbons, phenols, and other compounds. Among these, heterocyclic compounds accounted for the highest variety and content. Table 1 shows the GC-TOF/MS detection results of pyrazine compound contents in the four real malt samples. The main pyrazine compounds included pyrazine, 2,3-DMeP, 2,5-DMeP, 2,6-DMeP, TTMP, 3-ethyl-2,5-dimethylpyrazine, 2-ethylpyrazine, 2-methylpyrazine, and TMP. The total content of pyrazine compounds was correlated with the roasting degree of malt, following the order of PM < MM < CrM < CaM. Figure 5 shows the current response (ΔI) values of the constructed sensor for the detection of the four malt products, along with the GC-TOF/MS detection results of the pyrazine compound contents. The results indicated that CaM exhibited the highest current response value (108 ± 6 µA) with a total pyrazine content of 0.39 ± 0.02 mg/kg, while PM showed the lowest current response value (4.6 ± 0.2 µA) with a total pyrazine content of 0.092 ± 0.004 mg/kg. The current change of the biosensor was highly positively correlated with the pyrazine compound concentration, and the correlation coefficient was 0.96.

The results indicate that the olfactory biosensor can be used to specifically detect the total content of pyrazine compounds in real food products and accurately distinguish the differences in pyrazine contents among complex samples. Therefore, it has the potential for rapid, accurate, and reliable detection of pyrazine compounds in food.

### 3.7. Evaluation of the Olfactory Receptor Structure

Although approximately 400 human olfactory receptors have been identified, their crystal structures have not yet been fully elucidated [49,50]. Therefore, the 3D structure of the olfactory receptor protein OR5K1 was predicted using AlphaFold 3. AlphaFold 3 is a neural network-based model that exhibits performance comparable to experimental structures in most protein structure prediction tasks [51]. In addition, in the field of protein structure prediction, the AlphaFold3 model demonstrates superior performance compared with other homology modeling methods (e.g., SWISS-Model and I-TASSER), with an average accuracy exceeding 90% [52,53]. Furthermore, Figure S2B shows the evaluation results of the 3D model obtained from the SAVES website. The Ramachandran plot generated by the “PROCHECK” program can be used to calculate the percentage of residues in the most favored, allowed, and disallowed regions. Generally, a proportion exceeding 90% for all regions except the disallowed region usually indicates a high-quality protein model [54]. The Ramachandran plot revealed that 93.3% of the residues of OR5K1 were located in the most favored regions, 6.7% in the additionally allowed regions, and no residues were found in the generously allowed and disallowed regions. Thus, the OR5K1 model established by AlphaFold3 is reliable and suitable for subsequent molecular docking.

### 3.8. Molecular Docking Results Analysis

**Figure 6:**
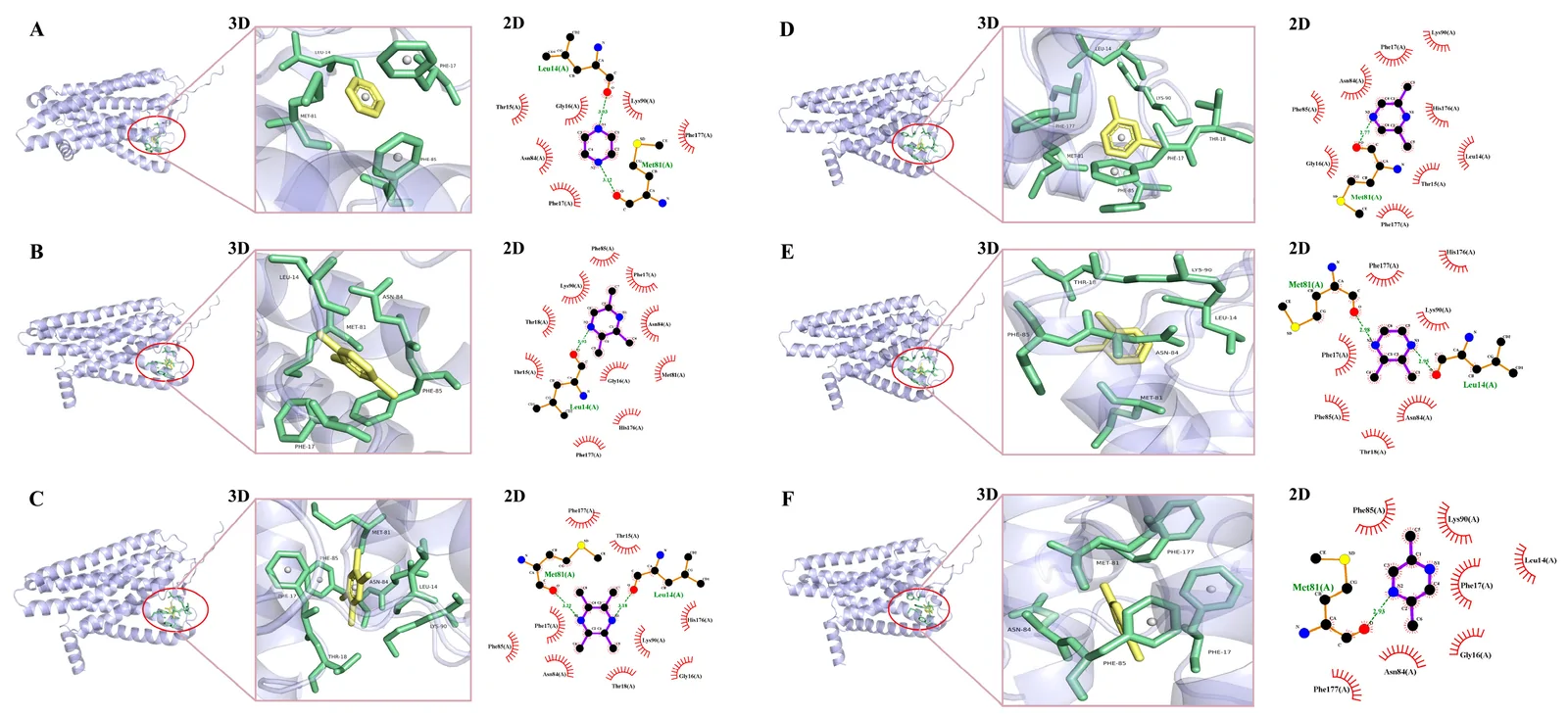
3D and 2D plots of optimal docking conformations between six pyrazine compounds and the olfactory receptor OR5K1. (**A**) OR5K1-pyrazine, (**B**) OR5K1-TMP, (**C**) OR5K1-TTMP, (**D**) OR5K1-2,6-DMeP, (**E**) OR5K1-2,3-DMeP, (**F**) OR5K1-2,5-DMeP.

The binding of odorant molecules to olfactory receptors initiates the transduction process that converts chemical stimuli into electrical signals, which represents the initial stage of olfactory perception. To elucidate the molecular interaction mechanism between pyrazine compounds and olfactory receptor OR5K1, molecular docking was performed (see Figure S2C and Figure 6). As shown in Figure S2C, the binding energies between OR5K1 and the six pyrazine odorants varied significantly, ranging from −3.9 to −6.0 kcal/mol. Binding energy is a critical indicator for judging the spontaneity of molecular interactions; a value less than zero indicates that the process occurs spontaneously [55]. These results confirm the spontaneous binding between OR5K1 and pyrazine odorants. Among these compounds, TMP exhibited the lowest binding energy of −6.0 kcal/mol, indicating the strongest affinity with OR5K1. In contrast, pyrazine showed the weakest affinity, with a binding energy of −3.9 kcal/mol. According to the findings of Zeng et al. [56], the binding energy between olfactory receptors and odorant molecules correlates with the molecular weight of the compounds. Therefore, the weakest affinity of pyrazine for the receptor might be attributed to its relatively low molecular weight (80.09 Da). This result is consistent with the electrochemical data of the sensor, where TMP elicited the highest current response signal and pyrazine induced the lowest. It is hypothesized that the minimal binding energy of TMP renders the receptor–ligand complex the most stable. Additionally, the three methyl groups in TMP may confer a more “compact” spatial configuration [57], enabling the formation of more hydrophobic interactions with the hydrophobic residues (Phe17, Phe85, Phe177, Leu14, Met81) in the binding pocket of OR5K1, thus resulting in a higher current response. It is worth noting that although 2,6-DMeP and 2,3-DMeP exhibited similar calculated binding energies (−5.1 kcal/mol for both), their electrochemical responses differed (Figure 4D). This observation suggests that the sensor response is influenced not only by the calculated binding affinity but also by other factors such as binding kinetics, molecular orientation, and mass transport efficiency, consistent with the multi-factor nature of electrochemical biosensor responses discussed above.

Figure 6 illustrates the interaction analysis between OR5K1 and pyrazine odorants, revealing that hydrogen bonds and hydrophobic interactions are two crucial forces driving the binding of odorant molecules to olfactory receptors, which is consistent with the findings reported in previous studies [58,59]. Furthermore, it was observed that the amino acid residues Leu14 and Met81 in OR5K1 are more likely to form hydrogen bonds with pyrazine odorants, with bond lengths ranging from 2.92 to 3.18 Å and 2.77 to 3.33 Å, respectively. Meanwhile, the residues Thr18, Asn84, Phe177, Phe17, Phe85, and Lys90 tend to mediate hydrophobic interactions with pyrazine odorants. These findings further demonstrate that the binding affinity between pyrazine odorants and OR5K1 is not solely determined by hydrogen bonds but is a synergistic effect of both hydrogen bonds and hydrophobic interactions. In the receptor–odorant interactions, the hydrophobic residues Tyr and Phe are present in relatively high proportions, which has also been observed in previous research [56,60]. These results underscore the critical role of hydrophobic amino acid residues and hydrophobic interactions in maintaining the structural stability of the odorant–receptor complex. Moreover, the benzene ring structure of pyrazine compounds facilitates π−π stacking interactions with the receptor, thereby further enhancing the binding stability between odorant molecules and olfactory receptors [61]. Nicoli et al. combined homology modeling and AlphaFold2 modeling and localized 2-ethyl-3,6-dimethylpyrazine (EC_50_ = 14.85 μM) to a hydrophobic core pocket formed by L104 ^3.32^ and L255 ^6,51^, confirming these two leucine residues as key binding sites in OR5K1 and supporting that pyrazine recognition by OR5K1 is governed by shape complementarity and hydrophobic stacking [16]. Zhu et al. utilized molecular docking to further identify the TM3 helix as a critical binding region for 2-ethyl-3,6-dimethylpyrazine, with hydrogen bonds, hydrophobic interactions, and π–π stacking as the primary forces stabilizing the receptor–ligand complex. Collectively, Leu, Met, Thr, Lys, Phe, and Asn constitute the essential amino acid residues mediating OR5K1–pyrazine recognition [62,63]. Collectively, these results indicate that the residues Leu, Met, Thr, Lys, Phe, and Asn are essential for promoting the binding of OR5K1 to pyrazine odorants.

### 3.9. In Silico Site-Directed Mutagenesis Results Analysis

To further verify the key amino acid residues identified in this study, in silico site-directed mutagenesis was performed. Table 2 presents the comparative results of the molecular interactions between six pyrazine odorants and the mutant olfactory receptor OR5K1. The results indicated that mutations of the key amino acid residues in OR5K1 generally reduced its binding affinity for pyrazine, TMP, and 2,5-DMeP. Among these, mutations of the six potential key amino acid residues in OR5K1 all decreased the binding affinity for TTMP, with the Leu14Ala mutation exerting the most significant effect, which caused the binding energy to change from −4.6 kcal/mol to −4.3 kcal/mol. The decrease in binding affinity might be attributed to the Leu14Ala mutation, which disrupted the hydrogen bonds between OR5K1 and TTMP, leading to the binding of OR5K1 with fewer potential key residues (Table 2). In addition, despite the decreased binding affinity, the interactions between the mutant OR5K1 and TTMP still involved the potential key residues. Therefore, the above results demonstrated that these six residues are the key binding sites for the interactions between OR5K1 and pyrazine odorants.

## 4. Conclusions

In this study, an electrochemical biosensor based on the olfactory receptor OR5K1 was successfully developed for the detection of pyrazine compounds. The integration of OR5K1 with the AuNP–PB/ZIF-8@SWCNT/Ti_3_C_2_ MXene nanocomposite enabled sensitive and selective detection of pyrazines in both standard solutions and real malt samples, demonstrating the potential of receptor-based biosensors for food flavor analysis. Molecular docking and in silico site-directed mutagenesis predicted that Leu14, Met81, Asn84, Phe17, Phe85, and Lys90 are key residues involved in pyrazine recognition, with hydrogen bonds, hydrophobic interactions, and π−π stacking as the primary driving forces. These computational findings provide a molecular basis for understanding the recognition mechanisms of pyrazine-related roasted aromas and may guide future structure-based screening of aroma compounds. Despite the promising results, several limitations should be acknowledged. The biosensor is currently only applicable for liquid-phase detection, and the identified binding residues and interaction mechanisms are derived from computational predictions that require experimental verification through in vitro and in vivo studies. Future work will focus on miniaturizing the biosensor into a portable device, expanding the selectivity evaluation to include other heterocyclic compounds such as pyridines and pyrroles, and validating its performance across a broader range of food matrices. With further optimization, this biomimetic sensing platform holds great potential for on-site, rapid, and accurate flavor analysis, contributing to intelligent quality monitoring and control in the food industry. Overall, this study not only provides a practical tool for pyrazine detection but also offers mechanistic insights into olfactory receptor–ligand interactions, paving the way for the development of next-generation bioelectronic sensors for food flavor analysis.

## Figures and Tables

**Table 1:** Pyrazines in four types of malt using GC-ToF/MS.

Compounds	Concentration (mg/kg)			
	MM	PM	CrM	CaM
2,3-dimethyl-Pyrazine	0.019 ± 0.001 ^ab^	0.018 ± 0.002 ^ab^	0.05 ± 0.01 ^b^	0.067 ± 0.003 ^a^
2,5-dimethyl-Pyrazine	/^c^	0.02 ± 0.01 ^b^	0.026 ± 0.01 ^a^	0.148 ± 0.015 ^ab^
2,6-diethyl-Pyrazine	0.016 ± 0.005 ^b^	/	0.017 ± 0.004a	0.01 ± 0.001 ^ab^
2,3,5,6-Tetramethylpyrazine	/	/	0.013 ± 0.004 ^ab^	0.027 ± 0.002 ^a^
Pyrazine	0.003 ± 0.0004 ^ab^	0.004 ± 0.0003 ^ab^	0.016 ± 0.003 ^a^	0.016 ± 0.001 ^b^
3-ethyl-2,5-dimethyl-Pyrazine	0.015 ± 0.003 ^ab^	/	/	0.028 ± 0.002 ^a^
2-Ethylpyrazine	/	/	0.037 ± 0.006 ^b^	0.067 ± 0.003 ^a^
2-Methylpyrazine	0.041 ± 0.004 ^b^	0.05 ± 0.01 ^ab^	/	0.028 ± 0.012 ^a^
2,3,5-Trimethylpyrazine	0.015 ± 0.008 ^ab^	/	0.066 ± 0.012 ^a^	/

^a,b^ different letters in the same row represent significant differences (*p* < 0.05). ^c^ “/” indicates that the compounds were not detected.

**Table 2:** Comparison results of molecular interactions between compounds and mutant OR5K1 via in silico site-directed mutagenesis.

Compounds	Receptors	Binding Energy (kcal/moL)	Residues Interacting with OR	
			Via H-Bonds and Bond Length (Å)	Via Hydrophobic Interactions
pyrazine	OR5K1	−3.9	Leu14(2.28); Met81(2.29)	—
	OR5K1-Leu14Ala	−3.8	Met81(2.19)	—
	OR5K1-Met81Ala	−3.9	Leu14(2.17)	—
	OR5K1-Phe17Ala	−3.7	Leu14(2.21); Met81(2.18)	—
	OR5K1-Phe85Ala	−3.8	Leu14(2.18); Met81(2.19)	—
TMP	OR5K1	−6.0	Leu14(2.08)	Phe17; Met85; Asn84; Phe85
	OR5K1-Leu14Ala	−5.9	—	Phe17; Ala81
	OR5K1-Met81Ala	−5.9	Leu14(2.12)	Phe17; Ala81; Asn84; Phe85
	OR5K1-Asn84Ala	−5.5	Leu14(2.15)	Phe17; Phe85; Phe177
	OR5K1-Phe17Ala	−5.4	Leu14(2.07); Met81(3.55)	Ala17; Asn84; Phe85
	OR5K1-Phe85Ala	−5.1	Leu14(2.71); Met81(2.29)	Thr18; Asn84; Ala85; Phe177; Lys90
TTMP	OR5K1	-4.6	Leu14(2.53); Met81(2.37)	Thr18; Asn84; Phe85; Lys90
	OR5K1-Leu14Ala	−4.3	Phe200(3.37); Tyr259(2.00)	Leu199; Val260; Leu264
	OR5K1-Met81Ala	−4.4	Leu199(3.59); Tyr259(2.12)	Val260; Leu264
	OR5K1-Thr18Ala	−4.6	Leu14(2.02)	Phe17; Ala18; Met81; Asn84 Lys90; Phe85; Phe177
	OR5K1-Asn84Ala	−4.4	Thr240(2.00) Tyr290(1.91)	Agr293; Asn294
	OR5K1-Lys90Ala	−4.4	Leu14(1.73) Met81(3.72)	Phe17; Thr18; Phe85; Phe177
	OR5K1-Phe85Ala	−4.6	Phe17(3.12) Met81(2.18)	Thr18; Lys24; Asn84; Lys90; Phe177
2,6-DMeP	OR5K1	−5.1	Leu14(2.82); Met81(2.07)	Phe17; Thr18; Lys90; Phe177
	OR5K1-Leu14Ala	−5.1	Met81(2.73)	Ala14; Phe17; Thr18; Lys90; Phe177
	OR5K1-Met81Ala	−5.0	Leu14(3.19)	Phe17; Lys90; Phe177
	OR5K1-Thr18Ala	−5.1	Leu14(3.07); Met81(1.91)	Phe17; Lys90; Phe177
	OR5K1-Lys90Ala	−5.1	Leu14(2.89); Met81(2.11)	Phe17; Thr81; Phe178
	OR5K1-Phe17Ala	−4.7	Leu14(2.96); Met81(1.99)	Ala17; Thr18; Lys90; Phe177
	OR5K1-Phe85Ala	−4.8	Leu14(2.05); Met81(2.53)	Phe17; Asn84; Ala85; Phe177
	OR5K1-Phe177Ala	−5.2	Leu14(2.86); Met81(2.13)	Phe17; Thr18; Lys90; Ala177
2,3-DMeP	OR5K1	−5.3	Leu14(2.13); Met81(2.15)	Thr18; Asn84; Phe85; Lys90
	OR5K1-Leu14Ala	−5.1	Met81(3.13)	Thr18; Asn84; Phe85; Lys90
	OR5K1-Met81Ala	−5.1	Leu14(2.16)	Thr18; Phe85; Lys90
	OR5K1-Thr18Ala	−5.3	Leu14(2.11); Met81(2.15)	Asn84; Phe85; Lys90
	OR5K1-Asn84Ala	−5.3	Leu14(2.19); Met81(2.24)	Thr18; Ala84; Phe85; Lys90
	OR5K1-Phe85Ala	−5.1	Leu14(2.15); Met81(2.30)	Thr18; Lys90
	OR5K1-Lys90Ala	−5.0	Leu14(2.00); Met81(2.45)	Phe17; Thr18; Asn84; Phe85
2,5-DMeP	OR5K1	−5.3	Met81(2.10)	Phe17; Asn84; Phe85; Phe177
	OR5K1-Met81Ala	−5.1	—	Phe17; Asn84; Phe85; Phe177
	OR5K1-Asn84Ala	−4.9	Met81(2.09)	Ala17; Asn84; Phe85; Phe177
	OR5K1-Phe17Ala	−4.7	Leu14(3.34); Met81(2.18)	Phe17; Asn84; Phe177
	OR5K1-Phe85Ala	−4.9	Met81(2.09)	Ala17; Asn84; Phe85; Phe177
	OR5K1-Phe177Ala	−5.2	Met81(2.24)	Phe17 Asn84 Phe85 Ala177

Note: All data related to mutagenesis are obtained from computational simulations, and no in vitro experiments were performed.

## Data Availability

The original contributions presented in this study are included in the article/Supplementary Material. Further inquiries can be directed to the corresponding authors.

## Supplementary Materials

The following supporting information can be downloaded at: https://www.mdpi.com/article/10.3390/foods15183355/s1, Figure S1: The amino acid sequence of the DJO1. Figure S2: (A) The Ramachandran plot. (B) The 3D structures of olfactory receptors OR5K1. (C) The binding energy between olfactory receptors and ligands. Table S1: The basic information of six common pyrazines. Table S2: Types and concentrations of VOCs in four types malts.
