# Supplementary material for: Development of an Olfactory Receptor (OR5K1)-Based Biosensor for Pyrazine Detection in Foods
Source: Foods. 2026 Sep 21;15(18):3355. doi: 10.3390/foods15183355 (PMC13606829; doi:10.3390/foods15183355)
Supplement: Supplementary file 1 [file foods-15-03355-s001.zip › foods-4480159-supplementary.pdf]

Figure S1. The amino acid sequence of the DJO1.

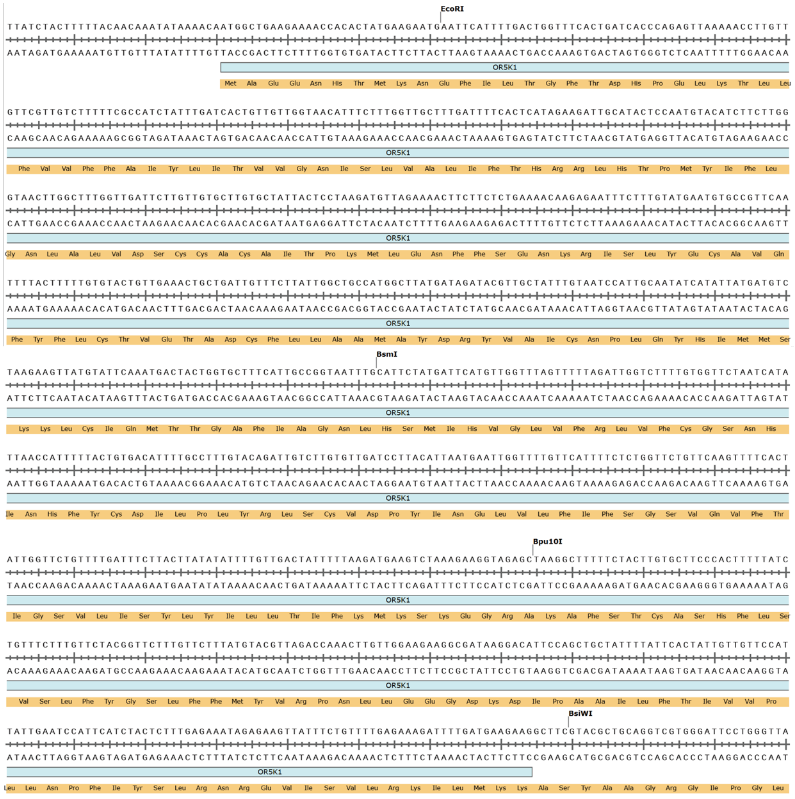

Figure S2. (A) The Ramachandran plot. (B) The 3D structures of olfactory receptors OR5K1. (C) The binding energy between olfactory receptors and ligands.

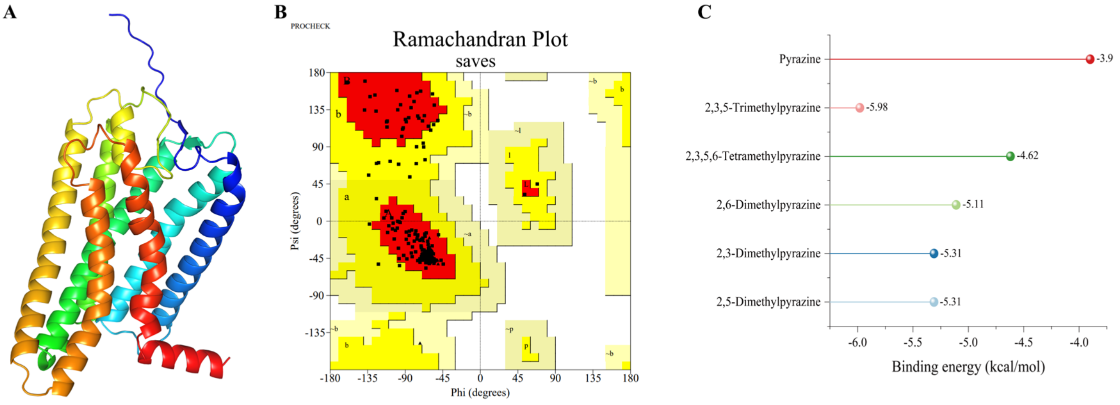

Table S1. The basic information of six common pyrazines.

Order	Full name	Abbrevia-tion	Molecular formula	Molecular weight	Boiling point ℃	Structure
1	pyrazine	-	$C_4H_4N_2$	80.09	115-116	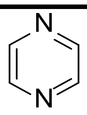
2	2,3,5-trimethylpyrazine	TMP	$C_7H_{10}N_2$	122.17	171-172	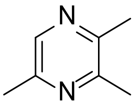
3	2,3,5,6-tetramethylpyrazine	TTMP	$C_8H_{12}N_2$	136.19	190.00	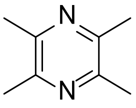
4	2,6-dimethylpyrazine	2,6-DMeP	$C_6H_8N_2$	108.14	154-155	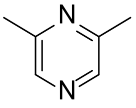
5	2,3-dimethylpyrazine	2,3-DMeP	$C_6H_8N_2$	108.14	155-157	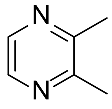
6	2,5-dimethylpyrazine	2,5-DMeP	$C_6H_8N_2$	108.14	154-156	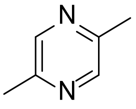

Physicochemical data in this table are from PubChem (https://pubchem.ncbi.nlm.nih.gov/).

Table S2. Types and concentrations of VOCs in four types malts.

No.	Compounds	CAS	Rt (s)	RI	Intensity (mg/kg)[b]			
				DB-WAX [a]	MM	PM	CrM	CaM
	Alcohols							
1	1,2,3-Butanetriol	4435-50-1	23.697	2213	0.059±0.006	/	/	/
2	1,2-Ethanediol	107-21-1	15.638	1593	0.45±0.01	0.7±0.1	0.27±0.07	/
3	1,3-Butanediol	107-88-0	17.343	1706	/	0.026±0.002	/	/
4	1-Hexanol	111-27-3	9.225	1333	0.024±0.001	0.067±0.005	/	0.010±0.001
5	1-Octadecanol	112-92-5	30.883	2557	/	0.79±0.56	/	0.036±0.009
6	1-Octen-3-ol	3391-86-4	12.201	1433	/	0.038±0.002	/	0.047±0.004

7	1-Pentanol	71-41-0	6.678	1235	0.038±0.002	0.092±0.001	0.056±0.009	0.193±0.011
8	1-Penten-3-ol	1576-96-1	5.039	1152	/	/	0.010±0.002	0.011±0.001
9	1-Propanol	71-23-8	3.683	1063	3.5±0.1	3.8±0.1	3.2±0.5	0.503±0.019
10	2-methyl-1-Propanol	78-83-1	4.165	1102	0.402±0.003	0.474±0.035	0.24±0.04	0.081±0.013
11	3-ethoxy-1-Propanol	111-35-3	9.871	1353	0.010±0.001	0.019±0.003	/	/
12	2-Butanol	18641-81-1	3.565	1052	/	/	0.12±0.13	0.016±0.003
13	Cyclopropyl carbinol	2516-33-8	21.357	2046	/	/	/	0.308±0.036
14	2,3-Butanediol	513-85-9	14.592	1553	7.174±0.132	1.244±0.081	1.023±0.311	0.048±0.004
15	Glycerin	56-81-5	24.668	2269	3.103±0.047	/	/	0.384±0.002
16	Propylene Glycol	57-55-6	15.110	1565	1.219±0.015	1.273±0.086	0.702±0.120	0.22±0.03
17	Triethylene glycol	112-27-6	25.032	2290	/	0.032±0.003	/	/
18	4-hydroxy-Benzeneethanol	501-94-0	41.861	2944	0.087±0.001	0.041±0.002	/	/
19	Phenylethyl Alcohol	22258	19.354	1871	0.113±0.004	0.056±0.001	0.044±0.007	/
20	2-Furanmethanol	98-00-0	16.121	1624	0.199±0.069	0.178±0.044	3.336±1.178	0.137±0.019
21	5-methyl-2-Furanmethanol	3857-25-8	17.018	1683	0.168±0.010	0.149±0.017	0.093±0.016	/
22	(Z)-2-Buten-1-ol	513-42-8	6.006	1206	0.147±0.068	0.078±0.028	0.041±0.028	0.006±0.0004
23	2-Propen-1-ol	107-18-6	4.378	1115	0.01±0.004	0.009±0.004	0.008±0.002	/
24	3-methyl-1-Butanol	123-51-3	5.863	1200	0.207±0.002	0.224±0.035	/	/
25	2-Butene-1,4-diol	110-64-5	19.896	1919	/	0.022±0.002	/	/
26	2-methyl-2-Propen-1-ol	513-42-8	8.838	1320	/	/	0.064±0.043	/
	Aldehydes							
27	2,3-dihydro-1-methyl-1H-Indole-5-carboxaldehyde	60082-02-2	20.381	1963	0.009±0.001	/	0.032±0.009	/
28	5-[(5-methyl-2-furanyl)methyl]-2-Furancarboxaldehyde	34995-74-9	25.803	2330		/	0.017±0.015	/

29	4-Oxohex-2-enal	20697-55-6	17.453	1715	0.011±0.0003	0.022±0.0001	/	0.046±0.004
30	5-Methyl-2-phenyl-2-hexenal	21834-92-4	21.228	2036	0.007±0.001	/	0.036±0.003	
31	Benzaldehyde	100-52-7	13.530	1485	0.026±0.001	0.02±0.001	0.029±0.004	0.05±0.003
32	2,4-dimethyl-Benzaldehyde	5779-94-2	18.151	1769	/	0.109±0.005	0.218±0.260	0.045±0.002
33	3-hydroxy-4-methoxy-Benzaldehyde	621-59-0	29.588	2505	/	/	/	0.042±0.004
34	4-hydroxy-Benzaldehyde	123-08-0	39.868	2877	/	/	0.102±0.014	/
35	α-ethylidene-Benzeneacetaldehyde	4411-89-6	19.559	1889	/	0.026±0.011	/	/
36	Furfural	35796	12.168	1432	0.99±0.69	1.01±0.54	8.64±1.70	0.377±0.003
37	Vanillin	121-33-5	29.607	2506	/	/	0.145±0.087	/
38	1H-Pyrrole-2-carboxaldehyde	1003-29-8	20.549	1979	0.025±0.003	0.005±0.001	0.135±0.017	0.190±0.013
39	(E)-2-methyl-2-Butenal	78508-96-0	29.831	2515	/	/	7.228±0.762	
40	Hexanal	66-25-1	4.049	1093	0.119±0.002	0.21±0.07	0.37±0.06	0.647±0.055
41	Heptanal	111-71-7	5.466	1177	/	/	0.035±0.014	0.021±0.004
42	Nonanal	124-19-6	10.352	1369	/	/	0.062±0.010	0.018±0.004
43	3-(2-furanyl)-2-Propenal	623-30-3	18.662	1810	/	/	0.020±0.002	/
44	(E, E)-2,4-Decadienal	25152-84-5	18.206	1773	/	/	1.455±0.177	0.235±0.044
45	5-Hydroxymethyl-furfural	67-47-0	28.284	2450	2.22±2.08	7.68±5.79	/	1.635±0.002
46	5-Hydroxy-2-pyridinecarbaldehyde	31191-08-9	45.384	3063	/	/	0.120±0.013	/
47	Hydroxy-Acetaldehyde	141-46-8	7.954	1290	/	/	11.55±4.40	0.035±0.006
	Ketones							

48	1,2-Cyclopentanedi-one	3008-40-0	17.619	1727	0.145±0.008	/	/	/
49	3-methyl-1,2-Cyclo-pentanedione	765-70-8	18.391	1788	/	/	0.14±0.02	0.030±0.003
50	2,5-Dihydroxypropio-phenone	938-46-5	24.167	2240	/	/	/	0.051±0.007
51	2-Acetyl-5-methylfu-ran	1193-79-9	15.344	1577	/	/	0.012±0.002	0.016±0.001
52	2-Heptadecanone	2922-51-2	23.589	2206	/	0.067±0.008	/	/
53	Dihydroxyacetone	96-26-4	21.258	2038	5.12±3.29	4.22±2.05	14.11±5.70	/
54	1-Hydroxy-2-buta-none	5077-67-8	9.543	1343	/	/	0.055±0.008	0.031±0.0001
55	2,3-Hexanedione	19322-27-1	21.707	2074	/	/	1.369±0.402	/
56	2,3-Pentanedione	600-14-6	3.769	1069	/	/	0.165±0.031	/
57	1-(1H-pyrrol-2-yl)-Ethanone	1072-83-9	19.985	1927	0.238±0.005	0.060±0.004	0.682±0.076	0.135±0.007
58	2-Heptanone	110-43-0	5.402	1173	/	/	/	0.044±0.003
59	5-hydroxy-2-Penta-none	1071-73-4	5.649	1187	/	/	0.051±0.032	/
60	(E, E)-3,5-Octadien-2-one	38284-27-4	13.651	1490	/	0.012±0.0003	0.013±0.002	/
61	3-Octen-2-one	1669-44-9	10.750	1381	/	/	/	0.045±0.008
62	Acetoin	513-86-0	7.330	1263	0.211±0.003	0.081±0.005	/	0.019±0.002
63	1-hydroxy-2-Pro-panone	116-09-6	7.615	1275	0.497±0.117	0.416±0.097	4.57±0.49	0.153±0.015
64	4-hydroxy-5-methyl-3(2H)-Furanone	19322-27-1	21.705	2074	0.604±0.137	0.347±0.046	/	/
65	1-(2-furanyl)-Etha-none	1192-62-7	13.202	1472	/	/	0.772±0.140	0.093±0.004
66	2-acetyl-Resorcinol	699-83-2	23.139	2176	/	/	/	0.072±0.011
67	Furyl hydroxymethyl ketone	17678-19-2	20.362	1962	0.281±0.104	/	0.03±0.004	0.213±0.030

68	Isophorone	78-59-1	15.041	1561	0.035±0.002	/	0.189±0.025	0.447±0.025
	Esters							
69	DL-Proline, 5-oxo-, ethyl ester	66183-71-9	31.098	2565	/	/	0.244±0.037	/
70	Glycine, N, N-dimethyl-, ethyl ester	33229-89-9	6.460	1226	/	/	/	0.103±0.011
71	Acetic acid, hydroxy-, ethyl ester	623-50-7	10.999	1389	0.333±0.004	0.408±0.034	0.042±0.014	0.016±0.001
72	Butanedioic acid, diethyl ester	123-25-1	16.407	1643	0.059±0.001	0.075±0.003	/	/
73	Butanoic acid, 2-hydroxy-, ethyl ester	52089-54-0	10.655	1378	/	0.067±0.004	/	/
74	Hexadecanoic acid, ethyl ester	628-97-7	24.001	2230	0.633±0.066	0.476±0.209	0.05±0.007	0.1±0.013
75	Hexadecanoic acid, methyl ester	112-39-0	23.355	2191	0.131±0.002	0.187±0.006	/	/
76	Methyl 2-furoate	17678-19-2	20.363	1962	/	0.293±0.074	/	/
77	Linoleic acid ethyl ester	2089036	29.491	2505	0.594±0.382	/	/	/
78	Octadecanoic acid, ethyl ester	111-61-5	28.043	2442	5.66±9.75	/	0.004±0.002	0.005±0.0003
79	Diethyl malonate	105-53-3	14.807	1548	0.007±0.001	0.006±0.002	0.021±0.001	0.005±0.0001
80	(E)--2-Butenoic acid, ethyl ester	623-70-1	5.105	1156	0.031±0.001	0.035±0.002	1.047±0.174	0.232±0.021
81	Propanoic acid, 2-oxo-, ethyl ester	617-35-6	6.919	1245	0.272±0.006	0.104±0.032	0.262±0.050	/
82	(L)-Propanoic acid, 2-hydroxy-, ethyl ester	687-47-8	8.793	1319	/	/	0.88±0.18	/
83	Benzenepropanoic acid, 3,5-bis(1,1-dimethylethyl)-	103929-83-5	31.229	2571	0.173±0.003	0.156±0.127	/	/
84	Ethanedioic acid, diethyl ester	95-92-1	12.802	1457	0.224±0.003	0.304±0.022	0.15±0.022	0.142±0.011

85	2-Hydroxy-gamma-butyrolactone	19444-84-9	22.366	2123	/	/	1.002±0.168	0.308±0.010
86	Propanoic acid, 2-hydroxy-, ethyl ester	97-64-3	8.796	1319	0.301±0.004	0.266±0.006	/	0.059±0.005
87	1-(acetyloxy)-2-Propanone	592-20-1	12.337	1439	/	/	0.272±0.041	/
	Acids							
88	3-methyl-2-Butenoic acid	541-47-9	17.960	1754		/	/	0.036±0.003
89	Lactic acid	6290-49-9	39.318	2859	16.078±0.729	/	0.188±0.063	/
90	N-Methylpyrrole-2-carboxylic acid	6973-60-0	41.078	2918	/	/	0.231±0.042	/
91	Palmitoleic acid	2416-20-8	40.674	2904	0.117±0.004	0.14±0.004	/	/
92	Pentadecanoic acid	1002-84-2	36.545	2764	0.058±0.018	/	/	/
93	Formic acid	64-18-6	13.205	1472	1.922±0.717	1.616±0.636	/	/
94	Acetic acid	64-19-7	11.984	1425	/	7.841±0.273	/	0.102±0.012
95	Benzeneacetic acid	103-82-2	29.562	2504	/	/	0.255±0.036	0.376±0.018
96	Propanoic acid	29102	14.025	1506	0.099±0.004	0.118±0.005	0.311±0.008	0.104±0.031
97	2-methyl-Propanoic acid	79-31-2	14.598	1538	0.086±0.071	/	/	0.088±0.007
98	Butanoic acid	107-92-6	15.624	1593	/	/	/	0.127±0.013
99	3-methyl-Butanoic acid	503-74-2	16.277	1634	0.143±0.003	0.109±0.003	/	0.399±0.042
100	Pentanoic acid	109-52-4	17.230	1697	0.029±0.001	/	0.041±0.006	0.014±0.001
101	4-methyl-Pentanoic acid	646-07-1	18.074	1763	0.014±0.001	0.012±0.002	0.55±0.11	0.555±0.067
102	4-oxo-Pentanoic acid	123-76-2	24.818	2278	/	/	0.434±0.064	/
103	Hexanoic acid	142-62-1	18.584	1803	0.229±0.013	0.516±0.006	/	0.105±0.012
104	Heptanoic acid	111-14-8	19.790	1910	/	/	0.141±0.011	0.057±0.006
105	Octanoic acid	623-66-5	20.996	2017	/	0.058±0.004		/
106	Nonanoic acid	112-05-0	22.365	2123	0.152±0.072	0.162±0.006	0.338±0.065	/
107	Tetradecanoic acid	544-63-8	33.564	2659	0.212±0.019	0.303±0.014		/

108	n-Hexadecanoic acid	21096	39.740	2873	/	11.837±6.445	0.957±0.139	/
109	Octadecanoic acid	21128	46.006	3084	1.574±0.057	/	0.539±0.207	/
110	(E)-9-Octadecenoic acid	112-79-8	47.016	3118	/	/	1.525±0.206	/
111	(Z,Z,Z)-9,12,15-Octadecatrienoic acid	463-40-1	50.476	3234	0.802±0.023	1.44±0.052	/	/
112	(Z,Z)-9,12-Octadeca-dienoic acid	60-33-3	48.463	3167	/	22.355±0.438	/	/
113	cis-11-Eicosenoic acid	5561-99-9	53.471	3335	0.173±0.052	0.139±0.010	/	/
114	Eicosanoic acid	506-30-9	52.526	3303	/	0.181±0.050	/	/
115	trans-13-Octadecenoic acid	693-71-0	46.836	3112	2.051±0.065	1.89±0.964	/	/
116	2-Propenoic acid	29135	15.713	1597	0.075±0.010	0.081±0.008	/	/
117	Benzoic acid	65-85-0	26.757	2378	0.066±0.010	/	0.027±0.006	0.006±0.002
	Heterocyclic Compounds							
118	5-methyl-2(3H)-Furanone	591-12-8	11.282	1398	0.044±0.010	/	1.5±0.191	0.147±0.008
119	2(5H)-Furanone	497-23-4	17.337	1705	0.312±0.084	0.321±0.0767	0.101±0.021	0.27±0.016
120	2-Vinylfuran	1487-18-9	5.153	1158	/	/	/	/
121	2,3-dihydro-Benzofuran	496-16-2	26.013	2341	0.088±0.006	0.086±0.006	/	0.314±0.030
122	2-pentyl-Furan	3777-69-3	6.262	1217	/	/	0.102±0.011	/
123	Furaneol	3658-77-3	20.707	1993	0.42±0.058	0.314±0.052	1.605±0.165	/
124	1-(1'-Pyrrolidinyl)-2-propanone	54151-38-1	11.669	1413	/	0.352±0.040	/	/
125	1-(2-furanylmethyl)-1H-Pyrrole	1438-94-4	18.326	1783	/	/	/	0.052±0.007
126	1-butyl-1H-Pyrrole	589-33-3	6.817	1241	0.01±0.001	0.009±0.006	/	/
127	2-ethyl-3,5-dimethyl-1H-Pyrrole	32990-59-3	17.714	1735	0.005±0.001	/	/	/
128	2-methyl-1H-Pyrrole	636-41-9	14.250	1518	0.02±0.003	/	/	/

129	3-ethyl-2,4-dimethyl-1H-Pyrrole	32990-59-3	17.715	1663	/	0.017±0.010	/	/
130	3-methyl-1H-Pyrrole	616-43-3	14.248	1590	/	0.022±0.009	/	/
131	Pyrrole	109-97-7	13.414	1481	0.021±0.0004	0.021±0.001	/	/
132	2-methyl-5-phenyl-Pyrrole	3042-21-5	30.539	2543	0.004±0.001	0.019±0.029	/	/
133	2(1H)-Pyridinone	109-00-2	26.594	2370	/	0.057±0.004	/	/
134	Niacinamide	98-92-0	40.486	2898	0.491±0.018	0.651±0.017	0.317±0.052	/
135	Picolinamide	1452-77-3	26.218	2351	/	/	0.009±0.003	/
136	Pyridine	110-86-1	5.479	1178	0.052±0.010	0.045±0.009	/	0.062±0.005
137	2-pentyl-Pyridine	2294-76-0	14.842	1550	/	/	/	/
138	2-propyl-Pyridine	622-39-9	14.843	1550	/	0.013±0.007	/	0.005±0.001
139	3-methyl-Pyridine	108-99-6	7.656	1277	0.014±0.001	/	0.058±0.005	0.106±0.012
140	2,3-dimethyl-Pyrazine	5910-89-4	8.939	1324	0.019±0.001	0.018±0.002	0.047±0.013	0.067±0.003
141	2,5-dimethyl-Pyrazine	123-32-0	8.473	1307	/	0.02±0.009	0.026±0.010	0.148±0.015
142	2,6-diethyl-Pyrazine	13067-27-1	11.650	1412	0.016±0.005	/	0.017±0.004	0.01±0.001
143	2,3,5,6-Tetramethylpy-razine	1124 - 11 - 4	10.279	1366	/	/	0.013±0.004	0.027±0.002
144	Pyrazine	290-37-9	10.290	1363	0.003±0.0004	0.004±0.0003	0.016±0.003	0.016±0.001
145	3-ethyl-2,5-dimethyl-Pyrazine	13360-65-1	11.992	1425	0.015±0.003	/	/	0.028±0.002
146	2-Ethylpyrazine	13925-00-3	8.561	1312	/	/	0.037±0.006	0.067±0.003
147	2-Methylpyrazine	109-08-0	6.958	1247	0.041±0.004	0.05±0.01	/	0.028±0.012
148	2,3,5-Trimethylpyra-zine	14667-55-1	10.730	1381	0.015±0.008		0.066±0.012	/
149	Butyrolactone	96-48-0	15.474	1584	0.897±0.007	0.505±0.029	0.328±0.047	/
150	l-Pantoyl lactone	5405-40-3	20.658	1989	/	/	/	0.152±0.014
151	Pantolactone	5405-40-3	20.658	1989	0.110±0.001	0.121±0.006	19.021±5.821	1.499±0.022
152	2-Pyrrolidinone	616-45-5	20.870	2007	0.467±0.004	0.292±0.044	0.578±0.091	0.454±0.039

153	Glutarimide	1121-89-7	25.784	2329	/	/	0.121±0.016	0.216±0.034
154	8-hydroxy-2H-1-Benzopyran-2-one	2442-31-1	44.151	3021	/	/	/	0.019±0.002
155	2H-Pyran-2,6(3H)-dione	5926-95-4	20.200	1947	/	0.076±0.017	/	/
156	5,6-dihydro-2H-Pyran-2-one	3393-45-1	18.686	1812	/	/	0.157±0.021	0.014±0.003
157	3-(Pyrrolidin-1-yl)cyclopent-2-en-1-one	36287-28-2	20.615	1985	/	0.011±0.002	/	/
158	4H-Pyran-4-one	108-97-4	18.261	1777	/	/	/	0.015±0.004
159	Hydantoin	461-72-3	51.037	3253	0.116±0.001	0.091±0.008	/	/
160	Indole	120-72-9	26.920	2386	/	0.077±0.004	/	0.065±0.006
161	Maltol	118-71-8	19.971	1926	0.373±0.048	0.402±0.008	16.227±1.459	0.969±0.057
162	4,5-dihydro-2-methyl-Oxazole	1120-64-5	4.900	1145	0.012±0.001	/	/	/
163	2-Furanone, 3,4-dihydroxytetrahydro	17675-99-9	42.703	2973	0.093±0.027	0.135±0.010	1.07±0.271	0.046±0.002
164	2,5-diformylfuran	823-82-5	20.013	1930	0.118±0.107	0.089±0.066	0.469±0.060	/
165	3-methyl-2,5-Furandione	616-02-4	16.671	1660	0.057±0.020	/	0.082±0.008	0.107±0.031
166	dihydro-3-methyl-2,5-Furandione	4100-80-5	19.456	1880	/	/	0.017±0.004	0.022±0.001
167	2-(8Z)-8-heptadecen-1-yl-4,5-dihydro-Oxazole	34900-26-0	36.413	2760	0.15±0.019	0.228±0.013	/	/
168	2,5-dimethyl-Pyrazine	123-32-0	8.473	1307	/	0.02±0.009	0.026±0.0010	0.148±0.015
	Hydrocarbons							
169	7-methyl-(E)-5-Undecene	57024-93-8	6.200	1214	/	0.083±0.024	0.117±0.072	/
170	1,2,4,5-tetramethyl-Benzene	95-93-2	11.155	1394	0.042±0.001	0.049±0.003	0.033±0.007	/
171	1-ethyl-4-methyl-Benzene	611-14-3	6.153	1212	0.029±0.001	0.031±0.002	0.005±0.001	/

172	pentamethyl-Benzene	700-12-9	15.812	1603	0.006±0.0001	0.008±0.0001	0.011±0.004	0.037±0.003
173	pentyl-Benzene	538-68-1	10.946	1387	/	/	0.047±0.007	0.014±0.0004
174	Decane	124-18-5	3.411	1039	/	0.017±0.003	/	/
175	Dodecane	112-40-3	6.039	1208	0.249±0.065	0.369±0.078	0.275±0.038	/
176	Heneicosane	629-94-7	21.991	2097	0.264±0.054	0.384±0.025	0.042±0.014	/
177	Naphthalene	91-20-3	17.152	1692	0.061±0.002	0.072±0.003	0.112±0.017	/
	Phenols							
180	2-methoxy-1,4-Benzenediol	824-46-4	40.443	2897	0.19±0.017	0.401±0.042	0.194±0.050	0.163±0.009
181	2-Methoxy-4-vinylphenol	7786-61-0	22.732	2148	0.183±0.007	0.203±0.011	0.214±0.029	/
182	Hydroquinone	13327-27-0	18.041	1760	/	/	0.066±0.007	/
183	Phenol	108-95-2	20.313	1957	/	0.066±0.021	/	0.1±0.009
184	2-methoxy-Phenol	32994	18.713	1814	/	/	/	0.098±0.006
185	2-methyl-Phenol	95-48-7	21.303	2042	/	/	/	0.011±0.001
186	4-ethenyl-2,6-dimethoxy-Phenol	28343-22-8	29.990	2522	0.03±0.024	0.065±0.003	0.015±0.002	/
187	4-ethyl-2-methoxy-Phenol	2785-89-9	20.633	1986	/	/	/	0.085±0.007
	Others							
188	2-methyl-N-(2-methylbutylidene)-1-Butanamine	35448-31-8	4.959	1148	0.01±0.006	0.331±0.081	/	/
189	2-Propenamide	29007	19.634	1896	/	/	0.027±0.003	/
190	Acetamide	60-35-5	17.642	1729	0.107±0.003	0.069±0.001	0.307±0.034	0.287±0.023
191	N-methyl-Acetamide	79-16-3	15.952	1613	/	/	/	0.022±0.012
192	Benzeneacetamide	103-81-1	33.621	2661	/	/	/	0.022±0.005
193	1,1-diethoxy-2-methyl-Butane	3658-79-5	3.926	1082	/	/	0.058±0.032	/
194	Cyclobutylamine	2516-34-9	31.724	1038	/	/	0.537±0.058	0.003±0

195	dl-Alanyl-l-leucine	1638-60-4	48.989	3184	/	/	/	0.142±0.022
196	Ethyl-1-propenyl ether	928-55-2	7.165	1256	0.013±0.003	/	/	/
197	Formamide	27735	17.838	1745	/	0.045±0.004	/	0.063±0.004
198	N,N-dimethyl-Formamide	25174	8.522	1311	0.014±0.002	0.024±0.001	/	0.055±0.005
199	1,1-diethoxy-Hexane	3658-93-3	6.435	1224	/	/	0.035±0.006	0.021±0.005
200	Hordenine	539-15-1	31.160	2568	3.589±0.085	4.426±0.218	4.219±0.448	/
201	N-L-alanyl-L-Alanine	1948-31-8	46.124	3088	/	/	/	0.161±0.006
202	Maleic anhydride	108-31-6	10.597	1377	0.041±0.006	0.026±0.009	/	/
203	N-Butyl-tert-butylamine	16486-74-1	18.845	1826	/	/	0.066±0.007	/
204	N-Ethyl-2-carbethoxyazetidine	54773-06-7	31.089	2565	/	0.077±0.010	/	/
205	4-methyl-Pentanamide	1119-29-5	21.124	2027	/	/	/	0.042±0.005
206	Phthalimide	85-41-6	38.987	2848	0.012±0.003	0.016±0.001	0.022±0.005	/
207	1,1-diethoxy-2-methyl-Propane	1741-41-9	3.327	1032	/	/	0.024±0.017	/
208	Succinic anhydride	108-30-5	21.192	2368	/	/	0.273±0.353	/
209	α-Furfuryliden-α-furylmethylamine	19377-82-3	23.568	2205	/	/	/	0.169±0.019
210	1,4-diethoxy-2-Butene	7250-85-3	26.202	2350	/	/	0.054±0.009	/
211	2,3-Diethoxy-propionic acid, ethyl ester	10120-24-8	18.474	1794	0.027±0.001	/	0.021±0.001	/

[a] The RIs were determined on an DB-WAX column using a standard mixture of n-alkane series (C_7 - C_{22}) as reference. [b] "/" indicating that the compounds were not detected.
